# Aberrant white matter and subcortical gray matter functional network connectivity associated with static and dynamic characteristics in subjects with temporal lobe epilepsy

**DOI:** 10.3389/fnins.2025.1571682

**Published:** 2025-05-14

**Authors:** Sukesh Kumar Das, George B. Hanna, Hai Sun, Bharat B. Biswal

**Affiliations:** ^1^Department of Biomedical Engineering, New Jersey Institute of Technology, Newark, NJ, United States; ^2^Department of Neurosurgery, Rutgers Robert Wood Johnson Medical School, New Brunswick, NJ, United States

**Keywords:** temporal lobe epilepsy (TLE), white matter (WM), resting state functional magnetic resonance imaging (rs-fMRI), dynamic functional connectivity (dFC), dynamic functional network connectivity (dFNC), functional connectivity strength (FCS)

## Abstract

Temporal lobe epilepsy (TLE) is a common type of epilepsy, with seizures primarily originating in the deep temporal lobe. This condition results in changes in connectivity across gray matter (GM), and white matter (WM) regions. This altered connectivity categorizes TLE as a network disorder, highlighting the need to investigate functional network connectivity (FNC) in WM areas. Dynamic functional connectivity (dFC) measures time-varying correlations between two or multiple regions of interest and derives clusters highlighting functional networks (FNs) where connectivity among regions behaves in a similar fashion. In this study, we included a total of 103 subjects from the Epilepsy Connectome Project, comprising 51 healthy controls (HC), and 52 subjects with TLE. We obtained static FNs (sFNs) and dynamic FNs (dFNs) using K-means clustering on ROI-based static functional connectivity (sFC) and dFC, respectively. Both static and dynamic FNCs were then separately investigated in HC and TLE subjects, with the latter demonstrating significant differences in WM networks. The static FNC was significantly decreased between the Forceps minor-Anterior corona radiata (ACR) - genu and left inferior longitudinal fasciculus (ILF) in TLE. Dynamic FNC significantly decreased between the corpus callosum (CC) (body) - superior corona radiata - right superior longitudinal fasciculus network and the Forceps minor - ACR - medial frontal gyrus network in subjects with TLE. This result implies that this WM connection changes with lower variability in TLE. On the other hand, the dynamic connections between the left temporal sub gyral - left thalamus - left pallidus - left hippocampus and right thalamus - right putamen - right temporal sub gyral - right pallidus network and the connections between the cingulum network and right thalamus - right putamen - right temporal sub gyral - right pallidus network significantly increased. These results indicate that these two GM subcortical connections change with higher variability in TLE. The study also demonstrates that the static functional connectivity strength (FCS) of the left ILF decreased significantly in subjects with TLE. However, the dynamic FCS of the splenium and brain stem were altered significantly in TLE, implying that the total dynamic connections of this network with all other networks experienced greater changes. Furthermore, the FNC suggests that the WM regions - ILF, superior and ACR, and CC exhibit connectivity changes related to the clinical features.

## Highlights

- Deriving Gray matter subcortical and white matter functional networks (FNs) by clustering ROI-based static and dynamic functional connectivity.
- Static and dynamic functional network connectivity (FNC) analysis in temporal lobe epilepsy (TLE).
- Static and dynamic functional connectivity strength (FCS) analysis in temporal lobe epilepsy (TLE).

## 1 Introduction

Temporal lobe epilepsy (TLE) is a chronic neurological disorder characterized by severe recurrent seizures and is the most common form of epilepsy in adults (Lariviére et al., 2020; Téllez-Zenteno and Hernández-Ronquillo, 2012). In TLE, the frequent seizures originate in the epileptogenic zone, typically within the medial temporal regions (Vlooswijk et al., 2010). The generation and propagation of these seizures result from hypersynchrony within the epileptogenic zone. Rivera Bonet et al. (2020) reported a loss of synchronization between the frontal and temporal regions associated with increased neuroticism. Additionally, cognitive impairment (Qin et al., 2020) and accelerated brain aging (Hwang et al., 2020) progress chronically in TLE. TLE can be conceptualized as a network disorder, with the epileptogenic zone acting as a critical node in the network (González Otárula and Schuele, 2020). Although TLE primarily affects the epileptogenic zones in the medial temporal regions, structural and functional changes have been shown to have widespread effects extending to limbic structures (e.g., the entorhinal and perirhinal cortices and the amygdala), subcortical regions (e.g., the thalamus), and neocortical regions (e.g., the frontal lobes and the superior, middle, and inferior temporal gyri), as evidenced by magnetic resonance imaging (MRI) and electroencephalogram (EEG) studies (Lieb et al., 1991; Spencer and Spencer, 1994; Spencer, 2002; Bartolomei et al., 2005; Lin et al., 2006; Bonilha et al., 2006; Bartolomei et al., 2004; Laufs et al., 2007; Haneef et al., 2012; Liao et al., 2010; Hermann et al., 1991; Giovagnoli, 2001; Mueller et al., 2004). The seizures are typically treated with antiepileptic medication or by resecting the epileptogenic zone (Thadani et al., 1995; Pittau et al., 2012). Therefore, it is crucial to identify the epileptogenic zone and understand the dysfunction of the networks affected by it.

Low-frequency fluctuations in the resting-state functional magnetic resonance imaging (rs-fMRI) signal are thought to reflect spontaneous neuronal activity (Biswal et al., 1995) via the blood oxygen level-dependent (BOLD) signal and have emerged as a means to study brain function. Functional connectivity (FC), obtained by assessing the synchronized activity between segregated brain regions, has been well-studied in different disorders (Fox and Raichle, 2007; Greicius, 2008; Lui et al., 2014). Functional networks (FNs) refer to groups of brain regions that work coherently to perform cognitive tasks or govern behavior, and these FNs are mostly obtained by data-driven methods such as independent component analysis (ICA) (Calhoun et al., 2001) or clustering (Ji et al., 2017). Using ICA, one can derive a series of spatially localized functional brain regions, including the Default Mode Network (DMN), Sensory Motor Network (SMN), Visual Network (VN), and so on, using BOLD time series. The clustering approach derives FNs by clustering the multivariate data, i.e., FCs. In traditional FC-based analysis, the measure is assumed to be stationary (static FC) over the entire fMRI scan. However, this is typically not the case, and time-varying measures, such as dynamic FC (dFC) analysis, are necessary. The temporal dependency among the FNs is called functional network connectivity (FNC). The correlation between the entire network time series leads to static functional network connectivity (sFNC), which represents the average FC between the FNs over the entire scan period (Jafri et al., 2008). In contrast, dynamic functional network connectivity (dFNC) measures the FC between the FNs over shorter periods, revealing the dynamic configuration of network connectivity (Allen et al., 2012).

The static FC (sFC) alteration has shown the association of different FNs in TLE (Liao et al., 2010; D'Cruz et al., 2019). Binder has reviewed fMRI studies in TLE, emphasizing the identification of the motor, language, and memory systems that are at risk in subjects treated surgically for intractable epilepsy (Binder et al., 2002). Subjects with TLE have been shown to exhibit slower processing speeds associated with decreased connectivity between the primary visual cortices and the left supplementary motor area, as well as between the right parieto-occipital sulcus and the right middle insular area using rs-fMRI (Hwang et al., 2019). Waites and colleagues have reported disrupted seed-based connectivity in the language area in left mesial TLE (mTLE) (Waites et al., 2006). Reduced connectivity within mesial temporal lobe structures and enhanced connectivity in the contralateral regions were observed in mTLE (Bettus et al., 2011). Studies reported decreased FC in the auditory, sensory, middle temporal, and dorsal attention networks, while increased FC was observed in the primary visual cortex and attention FNs in mTLE (Zhang et al., 2009a,b). Increased connectivity was observed in the left hippocampus and amygdala, while a decrease in connectivity was shown in the right lateral temporal lobe (Struck et al., 2021). The identification of the anterior temporal lobe structure, particularly the hippocampus, is highly suggestive of a syndrome-specific effect because these regions have long been associated with TLE (Engel, 1996). Pereira and colleagues demonstrated impairment within and between the hippocampus in unilateral mTLE (Pereira et al., 2010). Therefore, since TLE causes abnormal electrical activity that originates in the hippocampus and other nearby structures (Jerome Engel, 2001), researchers have focused on hippocampus-based static and dynamic FC analysis.

The dFC measure is useful for examining brain dysfunction in various mental disorders (Holtzheimer and Mayberg, 2011; Jones et al., 2012; Damaraju et al., 2014; Price et al., 2014; Choe et al., 2017; Zhang et al., 2022) because temporal variability, specifically the variance of FC, carries important information about brain states and can provide detailed insights into brain function. Alterations in networks have been associated with TLE using dFC (Morgan et al., 2020). The dFC between the hippocampus and the supplementary motor area, the pre- and post-central gyri, the cuneus, the middle occipital gyrus, and the superior frontal gyrus for seeding in the left and right hippocampus in TLE has also been shown to be greater than in controls (Laufs et al., 2014). Morgan et al. (2020) reported an increase in variance in the fMRI time series at the seizure focus in the hippocampus in subjects with mTLE. This may disrupt healthy FC dynamics and consequently decrease static hippocampal FC.

Functional organization and dysfunction have been studied using rs-fMRI in white matter (WM) across healthy controls (HC) and various disease groups (Ding et al., 2018; Peer et al., 2017; Wang et al., 2021; Ji et al., 2017; Jiang et al., 2019a,b; Cui et al., 2021; Li et al., 2021). A recent study has derived functional clusters based on dynamic functional connectivity (dFC), and the association between static functional connectivity (sFC) and dFC was estimated within WM and gray matter (GM). It was revealed that the WM functional networks (WM-FNs) are more dynamic in nature and contain rich spatiotemporal information, similar to that of GM (Wang et al., 2021). Based on previous studies, the network abnormalities in subjects with temporal lobe epilepsy (TLE) also included structural disturbances in the WM, which serve as the structural basis for carrying information to different regions of GM and account for almost half of the brain (Concha et al., 2009; Gross et al., 2006; Thivard et al., 2005; Chu et al., 2023, 2024). These studies anticipate identifying the widespread brain dysfunction of the WM networks using rs-fMRI associated with TLE. Most previous studies using rs-fMRI data from subjects with TLE have been conducted using the BOLD time course taken from GM regions, while the signal from WM has been neglected, except for a few studies (Jiang et al., 2019a,b; Cui et al., 2021; Li et al., 2021). Jiang et al. (2019b) demonstrated increased FC in the rolandic network and the pre/post-central network, along with decreased FC in the dorsal frontal network in unmedicated benign epilepsy with centrotemporal spikes compared with HC. Cui et al. (2021) highlighted the white matter functional network disorder in mesial TLE. When investigating functional abnormalities, the network that showed a significant difference was considered a seed, and the underlying relationship between region of interest (ROI) mean FC (based on the seed) and clinical variables (seizure frequency and duration of seizures) was investigated. The study reported increased ipsilateral deep WM connectivity with specific cortical regions: the insula, temporal lobe, and supramarginal gyrus. This finding reveals reduced connectivity in WM networks extending to extratemporal regions. The reported investigation only considered the sFC for clustering and identifying significant alterations. FCs among WM networks and between WM and GM networks were also investigated in unilateral TLE (Li et al., 2021). They reported decreased FCs among superficial WM networks and decreased FCs between WM networks and the hippocampus in the patient group. Though numerous studies, including those on the static and dynamic behavior of the BOLD response, have been conducted, it remains unclear which WM and subcortical GM circuits are most prone to alterations in TLE subjects. The derivation of clusters or FN and their subsequent analysis to identify altered circuits, based on their dynamic characteristics, may shed light on the fundamental mechanisms, informing us about declines in cognitive function due to recurrent seizures. Thus, an investigation of WM-FC alterations in a larger cohort using static and dynamic characteristics is necessary to understand brain function in TLE populations.

This study explores WM-FNs and alterations in FNC and functional connectivity strength (FCS) in subjects with TLE, focusing on the dynamic characteristics of FC. First, we derived WM-FNs using k-means clustering on the sFC and dFC. The k-means clustering technique groups similar connectivity patterns using dual features to provide a comprehensive understanding of distinct functional clusters by capturing both the average and time-varying relationships between brain regions. The sFC-based method groups ROIs into a cluster where the average connectivity is similar for all included ROIs. In contrast, the dFC-based method considers every ROI for inclusion in a cluster based on the changes in the FC of that ROI with all other ROIs over a shorter period, ensuring they are similar. After deriving these clusters, we analyzed their network connections (FNC) to determine which specific connections were significantly altered in subjects with TLE compared to HC. If the dFNC, with standard deviation as a statistic, between two networks is high, then the networks change diversely over time; otherwise, they change similarly. Since interictal epileptic discharges are often transient and spread across different brain regions, affecting multiple networks in epilepsy, dFNC analysis may provide additional insight into the underlying pathophysiological mechanisms in TLE (Klugah-Brown et al., 2018). Furthermore, we computed both static and dynamic FCS for each FN and identified those that exhibited significant alterations in FCS, providing information about the total connectivity changes of a derived FN with other FNs in TLE. The identified functional distinctions in WM were further examined to uncover the underlying connections between neurophysiological alterations and specific clinical features, particularly complex partial seizures and seizure frequency.

## 2 Method

### 2.1 Participants

We used publicly available data from the Epilepsy Connectome Project (ECP).^1^ The ECP study had a total of 236 subjects; for this study, we only used data sets from 196 subjects from the first session (Run 1) that had both T1 and resting-state fMRI data available. Out of 196 subjects, we considered 103 subjects (64 females and 39 males, average age 36.73 years) with 52 unilateral TLE (excluding those with bilateral or unrecognized-sided TLE) and 51 healthy controls (HC) to create balanced groups after excluding subjects based on framewise displacement. Out of the 52 TLE subjects, 34 and 18 subjects had their seizures on the left and right sides, respectively. The mean and standard deviation of the ages of the HC and TLE groups are 34.41 ± 11.10 and 37.28 ± 12.16. The Modified Edinburgh Handedness Quotient (MEHQ), ranging from −100 to +100, is presented in the histograms of the HC and TLE groups in the Supplementary material. If the MEHQ value is −100, then the subject is strongly left-handed, and if it is +100, then the subject is strongly right-handed. The mean and standard deviation of the MEHQ of the HC and TLE groups are 82.69 ± 31.23 and 77.04 ± 38.85, respectively.

### 2.2 Image acquisition

All the data from ECP subjects were acquired on standard GE Healthcare Discovery MR750 MRI systems (3T) housed at the Medical College of Wisconsin and the University of Wisconsin. The integrated body RF coil was used for excitation, while a Nova Medical 32-channel receive-only head coil was used for signal reception. T1-weighted structural images were acquired using a three-dimensional gradient-echo pulse (MPRAGE) sequence with the following parameters: repetition time (TR) = 604 ms, echo time (TE) = 2.516 ms, flip angle = 8 degrees, field of view (FOV) = 25.6 cm, voxel size = 0.8 mm (isotropic). Resting-state functional MRI (rs-fMRI) data were acquired using a gradient recalled echo (GRE) echo-planar imaging (EPI) sequence with the following parameters: TR = 802 ms, TE = 33.5 ms, FOV = 20.8 cm, flip angle = 50 degrees, number of slices = 72, voxel size = 2 mm (isotropic), multiband acceleration factor of 8. Two sessions, each containing a set of four 5-min resting state scans (axial acquisitions), were acquired in pairs of runs that alternated between anterior-to-posterior (AP) and posterior-to-anterior (PA) phase encoding directions, totaling eight scans. Participants were asked to gaze at a white cross on a black background. For every subject, we considered 361 time points in our study.

### 2.3 Data pre-processing

All the data across each subject was pre-processed as follows: (1) The first 10 volumes were discarded to ensure steady-state longitudinal magnetization; (2) The functional images were realigned; (3) Subjects with maximum displacements greater than 2 mm or 2 degrees were excluded from further analysis; (4) Individual T1 images were segmented into gray matter (GM), white matter (WM), and cerebrospinal fluid (CSF) to obtain the tissue probability map transformation from native to standard MNI space. The resulting segmented images were co-registered to functional space for each participant; (5) The nuisance signal (including 24 motion parameters: 6 rigid body head motion parameters at the current time point, 6 parameters at previous time points, and the 12 corresponding squared values) and the mean CSF time course were regressed out from all voxel time series; (6) Temporal filtering (using 5th-order Butterworth) was applied in the low-frequency range of 0.01–0.15 Hz to reduce non-neuronal contributions to BOLD fluctuations (Peer et al., 2017); (7) To minimize the mixing of GM and WM signals, individual functional images were spatially smoothed with an isotropic kernel of full width at half maximum (FWHM) of 4mm × 4mm × 4mm separately within the GM and WM masks (Peer et al., 2017). Then, GM and WM images were merged into full functional images; (8) Finally, the functional images were normalized to the standard MNI template with a voxel size of 2*mm* × 2*mm* × 2*mm*. The entire preprocessing task was performed using our in-house MATLAB scripts with SPM12^2^ in MATLAB R2023a.

### 2.4 Dataset and code availability

ECP data is publicly available at https://osf.io/exbt4/. The code for deriving the FNs is available at https://github.com/sentudas32/FNs.

### 2.5 Deriving WM networks using sFC

**Figure 1:**
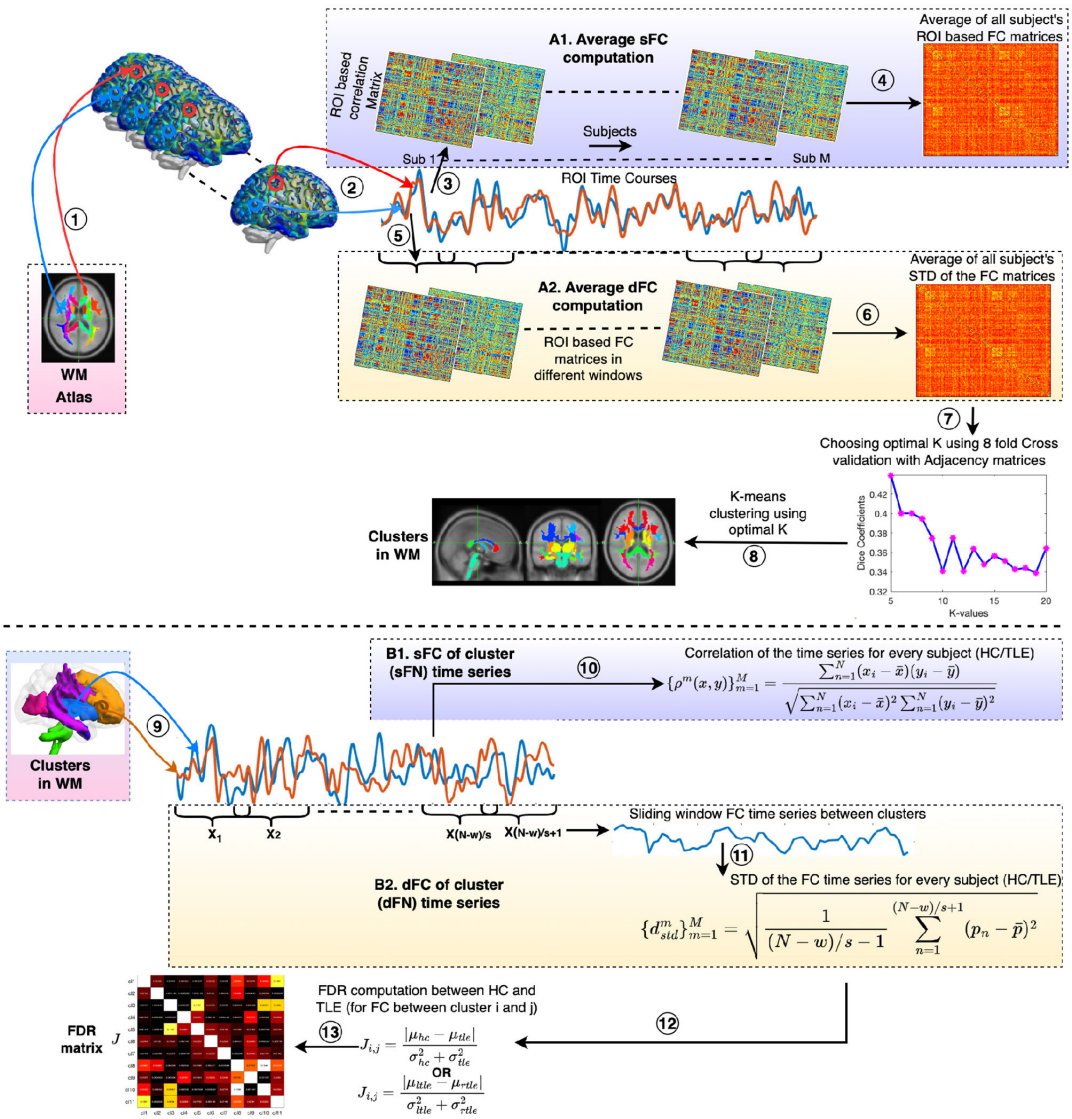
Procedure for clustering static and dynamic functional connectivity (sFC and dFC) to obtain static and dynamic functional networks (sFNs and dFNs) in WM and identifying discriminating FNs between HC and TLE (and lTLE and rTLE) using Fisher's discriminant ratio (FDR). The processing steps are as follows: (1) Time courses are extracted from ROIs of the atlas (Eve atlas). (2) Average ROI time courses were computed from WM regions. Panels A1 and A2 demonstrate the computation of average sFC and dFC, respectively. (3) Subject-wise ROI-based correlation (sFC) matrices were computed. (4) sFC matrices are averaged across all subjects (M), resulting in an average FC. (5) ROI-based FC computation is performed in every sliding window. (6) dFC with the standard deviation (STD) as a statistic across the windows was calculated. The average of the dFC matrices across all subjects was then computed. (7) A K-means clustering was performed on the averaged sFC or dFC matrix using correlation as the distance metric and with 50 replications. The optimal value of K was found using 8-fold cross-validation with adjacency matrices as described in Section 2.5. (8) K number of clusters (FNs) were derived from the K-means clustering using the optimal value of *K* with the lowest distortion from 100 replications. (9) Average time courses were extracted from all clusters (sFNs or dFNs) in WM. Panels B1 and B2 demonstrate the sFC and dFC of network time series, respectively. (10) FCs between the FNs were computed using Pearson's correlation for every subject (m = 1 to M) of either the HC or TLE groups. Here *x*_*i*_ and *y*_*j*_ are the BOLD responses of the clusters *x* and *y* at time points *i* and *j*, respectively. $\bar{x}$, ȳ, and *N* are the mean of the time courses of clusters *x*, *y*, and the total number of time points, respectively. Functional connectivities were Fisher's Z-transformed with sex and age effects regressed out. (11) FC time series were obtained using the sliding window method. All functional connectivities were Fisher's z-score transformed. The standard deviation of the FC time series was computed for every pair of clusters or dFNs for every subject (*m* = 1 to *M*) in the HC or TLE groups. Here, *p*_*n*_ is the correlation between two cluster time courses at the *n*_*th*_ slide and $\bar{p}$ is the average correlation across slides. *w* and *s* represent the length of the window and slide, respectively. Gender and age effects were regressed out from the dFCs. (12) FDR ratios were computed between the HC and TLE groups and between the left TLE (lTLE) and right TLE (rTLE) for every pair of FNs. (13) Finally, an FDR matrix was obtained across all pairs of FNs.

A schematic diagram for clustering the ROI-based sFC is shown in Figure 1, steps 1–4, 7, 8. A WM parcellation mask (Eve atlas, Type III WM parcellation map) (Oishi et al., 2009), consisting of 128 regions of interest (ROI), was used. This mask is a manual parcellation of 44 superficially located WM (SWM) and 56 deep WM (DWM) structures. The outline of the SWM is based on the 90% WM probability. As this parcellation includes the subcortical GM regions, including the thalamus, putamen (Clarke et al., 2022), hippocampus, and brainstem, and these regions are also found to be vulnerable to functional alterations in TLE (Hryniewicz et al., 2024; Lucas et al., 2023; Norden and Blumenfeld, 2002), we considered them in the clustering. Average ROI time courses were extracted from WM regions, and subject-wise Pearson's correlation matrices (sFC) were calculated; average correlation matrices were obtained across all subjects (i.e., M number of subjects including HC and TLE). Distinct WM functional networks were identified by performing k-means clustering on the average WM sFC matrices. In k-means clustering, the distance metric used was correlation, and 50 replications were taken into consideration. The number of clusters was chosen to be between 5 and 20. Cluster stability or the optimal number of clusters was achieved based on the *nf* (number of folds = 8) cross-validation using adjacency matrices (Peer et al., 2017). The average connectivity matrix was randomly divided into a given *nf* folds, and clustering computation was performed on each fold separately. The cluster indices were divided into chunks (chunk size, *cs* = 30) in each of the *nf* folds. For every pair of chunks, a binary 3D matrix (*cs* × *cs* × *nf*) was formed. Then, an averaged Dice coefficient was computed by comparing all *nf* adjacency matrices for every *K* with 50 replications. The *K* that yielded the highest Dice coefficients was the optimal *K*, deriving the stable clusters. Finally, *K*-means clustering was performed using the optimal number of *K*, and clusters (FNs) were obtained with the lowest distortion from 100 replications.

#### 2.5.1 sFC among the network time series and FDR analysis

An average of the time series of all voxels within every WM sFN was computed. The sFNs were defined by the resulting clusters from the K-means clustering, which produced *K* time courses for each of the subjects in the WM regions. Then, for each subject (HC or TLE), *K* cluster time courses from the sFNs are extracted. For each pair of network time courses, an sFC value is obtained using Pearson's correlation. The sFCs were Fisher's Z-transformed, and sex and age effects were regressed out for all subjects. Lastly, a static FNC (sFNC) matrix (*K* × *K*) was obtained for each subject, as depicted in Figure 1, steps 9, 10, 12, 13. Once these sFNC matrices were established, the Fisher discriminant ratio (FDR) was used to draw a discriminant assessment between two individual groups: first for comparing HC to the TLE group and again for comparing lTLE with rTLE (FISHER, 1936; Das et al., 2024). FDR is a measure used to assess the separability of classes or groups in pattern classification problems. It is the ratio of between-group variance to within-group variance. In our study, FDR values indicate which connectivity between the networks is most altered (or discriminating) between the two groups. A high FDR indicates that the two groups are widely separated in connectivity. The FDR between HC and TLE for each pair of clusters or sFNs *i* and *j* is as follows:


(1) $$J_{i,j}=\frac{\left|\mu_{HC}-\mu_{TLE}\right|}{\sigma_{HC}^{2}-\sigma_{TLE}^{2}},$$

, where μ_*HC*_ and $\sigma_{HC}^{2}$ are the mean and variance of the static connectivity scores of the paired sFNs of HC, respectively. μ_*TLE*_ and $\sigma_{TLE}^{2}$ are the mean and variance of the static connectivity scores of the paired sFNs of TLE, respectively. Similarly, the FDR between lTLE and rTLE for every pair of clusters or sFNs *i* and *j* is


(2) $$J_{i,j}=\frac{\left|\mu_{lTLE}-\mu_{rTLE}\right|}{\sigma_{lTLE}^{2}-\sigma_{rTLE}^{2}},$$

, where μ_*lTLE*_ and $\sigma_{lTLE}^{2}$ are the mean and variance of the static connectivity scores of the paired sFNs of lTLE, respectively. μ_*rTLE*_ and $\sigma_{rTLE}^{2}$ are the mean and variance of the static connectivity scores of the paired sFNs of rTLE, respectively (can be seen in Figure 1, steps 9, 10, 12, 13). These calculations result in an FDR matrix being obtained for all pairs of sFNs.

#### 2.5.2 Group difference in static functional network connectivity in WM

The synchronous activation of sFNs was assessed using two-sample t-tests between the sFNCs of two groups to identify significant connections. sFCs were first Fisher's Z-score transformed, and gender and age effects were regressed out, and two sample t-tests were performed between the HC and TLE groups, HC and lTLE groups, HC and rTLE groups, and lTLE and rTLE groups. The *p*-values were false discovery rate (*FDR*) corrected using the Benjamini and Hochberg method (Benjamini and Hochberg, 1995). Lastly, the significantly (*p* < 0.05) altered connections were illustrated.

#### 2.5.3 Functional connectivity strength using static functional network connectivity

The WM sFNs obtained from K-means clustering are used to quantify the degree of dysfunction in TLE compared to HC. The functional alteration was estimated utilizing FDR analysis and connectivity among WM-FNs, as described in Section 2.5.1 and Section 2.5.2, respectively. The static FNs that reveal significant alterations in terms of FCS were further analyzed. A two-sample *t-*test was performed between the static FCS (sFCS) of the HC and TLE groups to pinpoint the location of the alteration. The sFCS of the WM-network *i* is given by the following equation:


(3) $$\begin{array}{l} sFCS\left(sWM_{i}\right)=\overset{K-1}{\underset{j}{\sum }}sFNC_{\left(j,i\right)}, \end{array}$$

where, *sFNC*_(*j, i*)_ represents the sFC between *j*^*th*^ sWM-network and ith sWM-network. T-statistics and p-values from the t-tests were considered when investigating dysfunction. *p*-values were *FDR*-corrected using the Benjamini-Hochberg method. A similar micro-level analysis was also conducted between the subjects with lTLE and rTLE.

### 2.6 Deriving WM networks using dFC

dFC was computed to quantify temporal fluctuations using the sliding window approach with a window size of *w* samples and a slide of *s* samples (Wang et al., 2021; Chang and Glover, 2010; Sakoğlu et al., 2010; Kiviniemi et al., 2011; Handwerker et al., 2012; Hutchison et al., 2013). To employ the dynamic characteristics of FC, the same WM parcellation mask (Eve atlas), consisting of 128 equal-sized regions of interest (ROI), was used. The average time series of the ROIs from the atlas were extracted. FC among the ROIs was computed from each window. The temporal variability of the FCs was computed as the standard deviation of the matrices. The resulting connectivity matrix encoded how widely the FC fluctuated over time. The average standard deviation of the windowed FC matrices across all subjects (including HC and TLE) was obtained and is referred to as the group-level dFC. The WM dynamic functional networks (dFNs) were obtained by performing K-means clustering on the group-level dFC. During the K-means clustering, the correlation was used as the distance metric, and 50 replications were considered. The number of clusters was chosen from 5 to 20. The optimal number of clusters (*K*) was obtained using *nf* (number of folds = 8) cross-validation and a chunk size (*cz* = 30). The value of *K* that maximized the dice coefficient between adjacency matrices, as described in Section 2.5, was determined to be the optimal value of *K*. Finally, the clusters or dFNs were obtained using the optimal *K* value with the lowest distortion from 100 replications. Figure 1, steps 1, 2, 5, 8 demonstrate the computation of the dFNs.

#### 2.6.1 dFC among the network time series and FDR analysis

Average time courses of all voxels within the WM-dFNs were extracted, resulting in K-clustered (dFN) time courses. *K* time courses from the dFNs were extracted for each subject (HC or TLE). For each pair of dFNs, a dFC (standard deviation as a statistic) value was obtained using the sliding window method, resulting in a dFNC matrix (*K* × *K*) for each subject, as depicted in Figure 1, steps 9, 11. For dFNC computations, the window was slid by *s* samples, and the same analysis was repeated. All windowed FCs were Fisher's Z-score transformed, and one dynamic connectivity score (standard deviation) was obtained over time. Age and gender effects were regressed out from the dFCs for all subjects. A Fisher discriminant ratio (FDR) between the HC and TLE groups and between the lTLE and rTLE groups for every pair of clusters or dFNs was computed, following Equations 1, 2, respectively (Figure 1, steps 12, 13). For all pairs of dFNs, an FDR matrix (*J*) was obtained.

#### 2.6.2 Group difference in dynamic functional network connectivity in WM

The WM-dFNs, obtained from K-means clustering, were used to quantify the degree of diverse variations in TLE from HC or lTLE from rTLE by means of FDR analysis, as described in Section 2.6.1. The network time courses were then extracted to compute the dFC between the networks. This dFNC signified how similarly two functional networks change. If the dFC between two FNs was high, it implied that they changed similarly; otherwise, they changed diversely. This degree of variability is used to perform two-sample t-tests between two groups, and significant connections are identified. Here, all windowed FCs were Fisher's Z-score transformed. dFCs are obtained by considering standard deviation as the dynamic characteristic, with gender and age effects being regressed out from the dFC. Two-sample *t*-tests were performed between the HC and TLE groups, HC and lTLE groups, HC and rTLE groups, and lTLE and rTLE groups. The *p*-values were *FDR*-corrected using the Benjamini-Hochberg method, and significantly (*p* < 0.05) altered connections were illustrated.

#### 2.6.3 Functional connectivity strength using dynamic functional network connectivity

The WM dFNs were derived from K-means clustering and were used to quantify the total variability of a network with respect to other networks. This total variability was obtained by computing the dynamic FCS (dFCS). Alterations between the TLE and HC groups were also investigated using the dFCS. A two-sample *t*-test was performed between the dFCS of the HC and TLE groups to identify the alteration. The dFCS of the WM-network *i* is defined as follows:


(4) $$\begin{array}{l} dFCS\left(dWM_{i}\right)=\overset{K-1}{\underset{j}{\sum }}dFNC_{\left(j,i\right)}, \end{array}$$

, where *dFNC*_(*j, i*)_ represents the dFC between WM-network *j* and WM-network *i*. Here, all the windowed FCs were Fisher's Z-scored transformed, and dFCs were computed using standard deviation, followed by the regression of gender and age effects. T-statistics and *p*-values from the two-sample t-tests were used to investigate dysfunction. *p*-values were *FDR*-corrected using the Benjamini-Hochberg method, and significant dFNs were identified. A similar analysis was then carried out to compare the subjects with lTLE and rTLE.

### 2.7 Relationship between the FNCs and clinical features

A correlation analysis was performed to explore the underlying relationships between the FNCs (static and dynamic) and the clinical variables for each pair network (FN). For each significant pair of FNs, which was identified using either the FNCs analysis or the FDR analysis, the FCs were extracted from the interacting networks for every subject with TLE (left or right) and correlated with the monthly frequency of complex partial seizures (CP Freq) and the number of seizures captured (EEGSzCount). The correlation values were then analyzed. Information on CP Freq was only available for 35 subjects, and EEEGSzCount data was available for 47 subjects.

## 3 Results

### 3.1 WM networks using sFC and dFC

**Figure 2:**
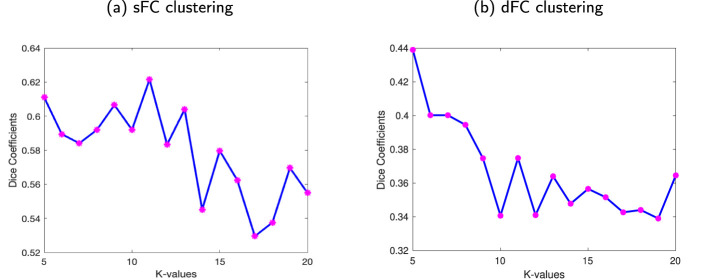
Stability of the clustering FC for different numbers of clusters. The average Dice coefficient of the clustering solutions (adjacency matrices) for each number of clusters ranging from *K* = 5 to 20. **(a)** Dice coefficients from clustering sFC. **(b)** Dice coefficients from clustering dFC. For both methods, the optimal value of *K*, i.e., *K* = 11 for WM, was found using 8-fold cross-validation with adjacency matrices.

**Figure 3:**
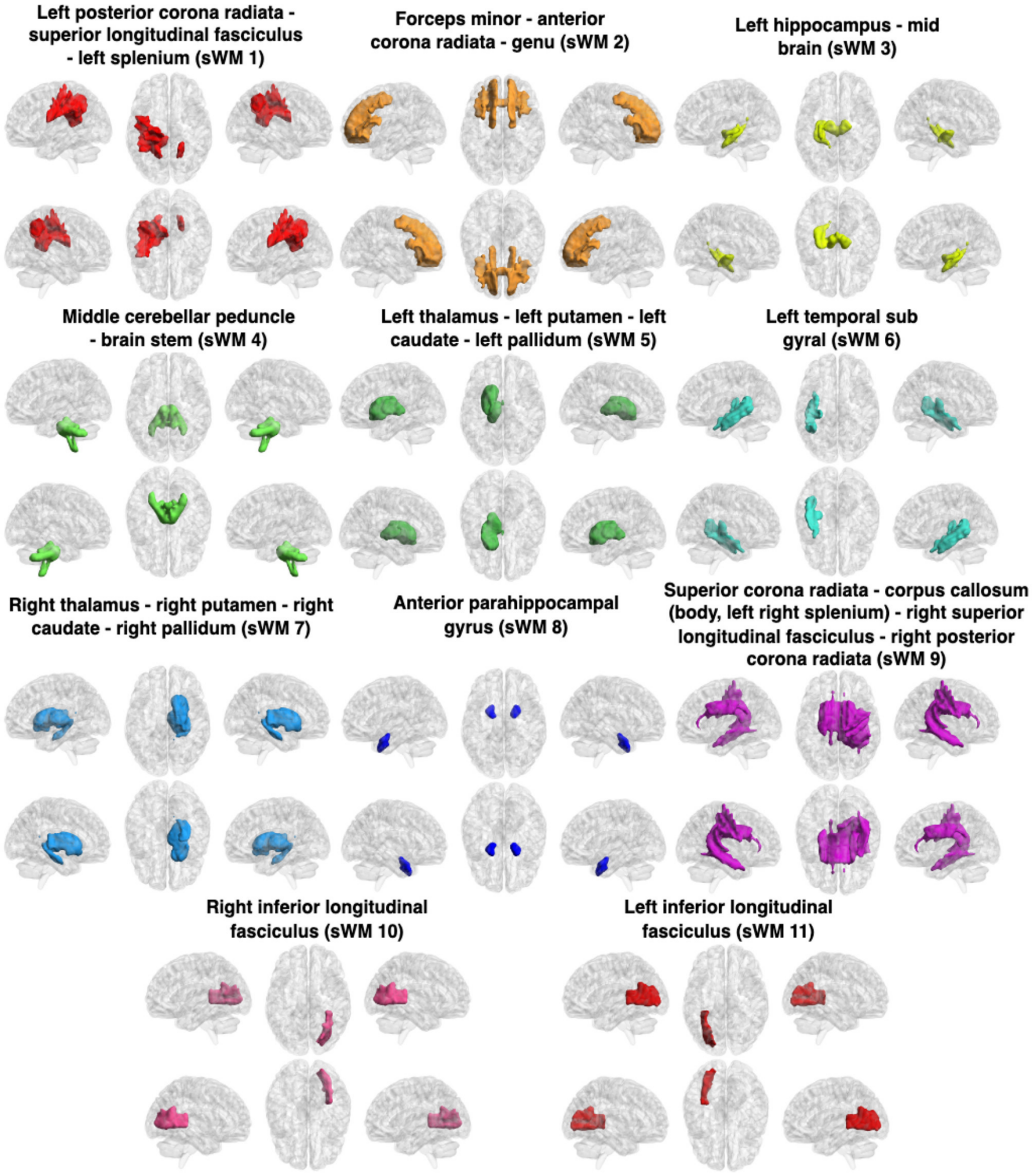
Clusters (sFNs) obtained from K-means clustering of the sFC in WM. The WM networks (sFNs) are, from left to right and top to bottom: lPCR - lSLF - CC(splenium) (sWM1), FM - ACR - CC(genu) (sWM2), lHC - MB (sWM3), MCP - BS (sWM4), lT - lP - lC - lPa (sWM5), lTSG (sWM6), rT - rP - rC - rPa (sWM7), APHCG (sWM8), SCR - CC (body, left splenium) - rPCR (sWM9), rILF (sWM10), and lILF (sWM11). All details of the abbreviations for the regions are provided in Table 1.

**Figure 4:**
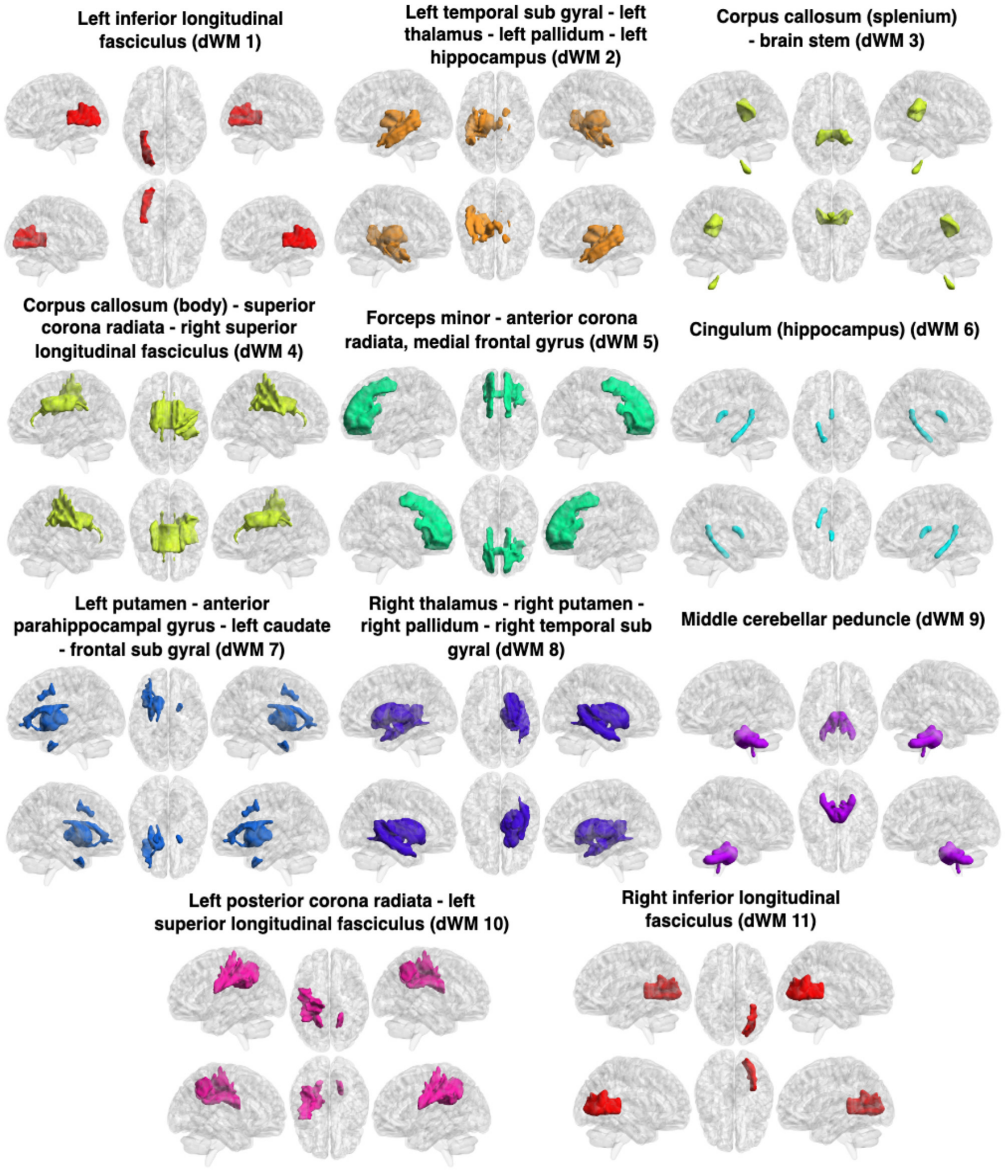
Clusters (dFNs) obtained from K-means clustering of the dFC in WM. The WM networks (dFNs) are, from left to right and top to bottom: lILF (dWM1), lTSG - lT - lPa - lH (dWM2), CC (splenium) - BS (dWM3), CC (body) - SCR - rSLF (dWM4), FM - ACR - MFG (dWM5), cingulum (hippocampus) (dWM6), lP - APHCG - lC - FSG (dWM7), rT - rP - rTSG - rPa (dWM8), MCP (dWM9), lPCR - lSLF (dWM10), and rILF (dWM11). All details of the abbreviations for the regions are provided in Table 1.

The WM sFNs were obtained by performing K-means clustering approaches on atlas-based average sFC in WM. The optimal number of clusters, i.e., *K*, was achieved by maximizing the Dice coefficient between the adjacency matrices using 8-fold cross-validation. The Dice coefficients for different numbers of clusters have been shown in Figure 2a. It can be observed that the Dice coefficient attained its highest value (0.622) at *K* = 11. The optimal value of *K* = 11 was used to derive the final clusters or sFNs. The resulting *K* clusters are presented in Figure 3. The WM-dFNs were obtained by performing K-means clustering on average ROI-wise dFC within the WM parcellations. The optimal number of clusters was also obtained by maximizing the Dice coefficient between the adjacency matrices in 8-fold cross-validation. Figure 2b demonstrates the Dice coefficients with different values of *K*. The Dice coefficient at the optimal *K*(= 11) value is 0.375. The Dice coefficient was much lower in dFC-based clustering than in sFC-based clustering due to the dFC encoding of the standard deviation, which extends to a much lower range than that of sFC (Pearson correlations). The resulting clusters obtained from the K-means clustering of the dFC are presented in Figure 4.

#### 3.1.1 Static and dynamic functional networks in WM and FDR

**Figure 5:**
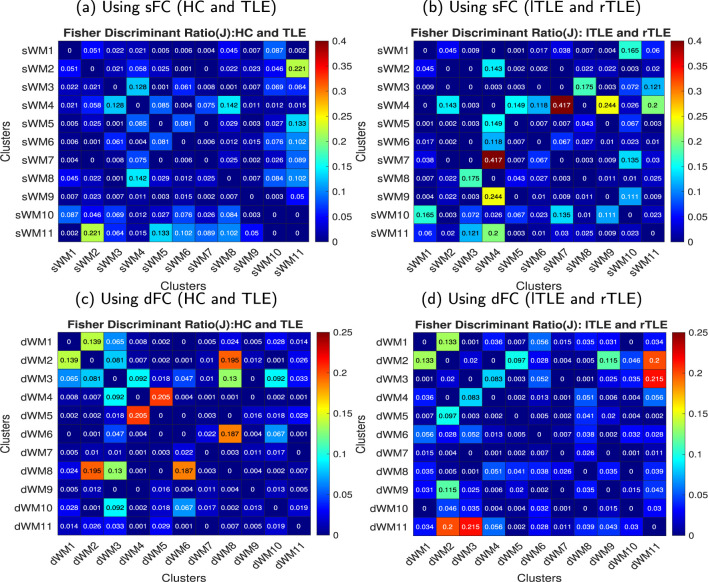
FDR matrices between HC vs. TLE and lTLE vs. rTLE using the sFC and dFC, respectively. Every element in the matrix was obtained for a pair of networks (sFNs or dFNs), and the FDR matrix was computed across all pairs of networks. **(a)** FDR matrix between HC and TLE using sFC, **(b)** FDR matrix between lTLE and right rTLE using sFC, **(c)** FDR matrix between HC and TLE using dFC, and **(b)** FDR matrix between lTLE and right rTLE using dFC in the WM. **(a)** Using sFC (HC and TLE). **(b)** Using sFC (lTLE and rTLE). **(c)** Using dFC (HC and TLE). **(d)** Using dFC (lTLE and rTLE).

The obtained sFNs using clustering of the sFCs in WM have been demonstrated in Figure 3. The sFNs include the following networks: sWM1, sWM2 ….sWM11. The sFNs and regions lying within the networks are also shown in Table 1. FDR was computed using interactions between network (sFN) time series. The FDR matrix between HC and TLE is demonstrated in Figure 5a. The connectivity between sWM2 and sWM11 (sWM2–sWM11) attained an FDR value of 0.221, revealing the highest alteration between the TLE and HC groups in the WM region. Another interaction of WM-sFNs, sWM4 and sWM8 (sWM4–sWM8), attained an FDR value of 0.142. The FDR matrices between lTLE and rTLE using sFC in the WM regions are demonstrated in Figure 5b. It can be observed that the interaction between sWM4 and sWM7 (sWM4–sWM7) revealed higher alteration (0.41) compared to the FDR values between HC and TLE, implying greater separability between lTLE and rTLE. In contrast, the dFNC-driven FDR exhibited higher values (0.43) for HC and rTLE, suggesting more dynamic changes between HC and rTLE (can be seen in Supplementary material). Interactions of sWM4–sWM9 and sWM4–sWM11 demonstrate alterations in WM, producing the FDR values of 0.244 and 0.2, respectively, when discriminating between lTLE and rTLE (Figure 5b). Figure 4 demonstrates 11 WM-dFNs obtained from clustering the WM parcellations based on stability characteristics. The dFNs and regions falling within the corresponding networks are also shown in Table 1. Using dFC, FDR was computed using interactions between the cluster time series, as described in the method Section 2.6.1. The FDR matrices between HC and TLE and lTLE and rTLE using dFC are demonstrated in Figures 5c, d, respectively. The connectivity between the clusters dWM4 and dWM5 (dWM4 - dWM5) attained the highest FDR value of 0.205 between HC and TLE, revealing the highest alteration in the WM region using dynamic characteristics. Other interactions of WM-dFNs, i.e., dWM2–dWM8 and dWM6–dWM8, attained the FDR values of 0.195 and 0.187, respectively. The WM-dFNs can find alteration between left and right TLE in terms of the network interactions. The network pairs, dWM3–dWM11 and dWM2–dWM11, reveal FDR values of 0.215 and 0.20, respectively, and can find the alterations in the left TLE from the right TLE.

**Table 1:** All static and dynamic functional networks (sFNs/dFNs) and the regions corresponding to the FNs in the brain. The abbreviations of the regions are also given.

	**WM networks**	**Regions in the brain**
	sWM1	Left posterior corona radiata (lPCR), left superior longitudinal fasciculus (lSLF),
		left corpus callosum (splenium) (CC(splenium))
	sWM2	Forceps minor (FM), anterior corona radiata (ACR), CC (genu)
	sWM3	Left hippocampus (lHC), midbrain (MB)
	sWM4	Middle cerebellar peduncle (MCP), brain stem (BS)
	sWM5	Left thalamus (lT), left putamen (lP), left caudate (lC), left pallidum (lPa)
sFNs	sWM6	Left temporal sub gyral (lTSG)
	sWM7	Right thalamus (rT), right putamen (rP), right caudate (rC), right pallidum (rPa)
	sWM8	Anterior parahippocampal gyrus (APHCG)
	sWM9	Superior corona radiata (SCR), CC (body, left splenium), superior longitudinal fasciculus (SLF),
		right posterior corona radiata (rPCR)
	sWM10	Right inferior longitudinal fasciculus (rILF)
	sWM11	Left inferior longitudinal fasciculus (lILF)
	dWM1	Left inferior longitudinal fasciculus (lILF)
	dWM2	Left temporal sub gyral (lTSG), left thalamus (lThalamus), left pallidum (lPa), left hippocampus (lH)
	dWM3	Corpus callosum (splenium) [CC(splenium)], brain stem (BS)
	dWM4	Corpus callosum (body) [CC(body)], superior corona radiata (SCR),
		right superior longitudinal fasciculus (rSLF)
	dWM5	Forceps minor (FM), anterior corona radiata (ACR), medial frontal gyrus (MFG)
dFNs	dWM6	Cingulum (hippocampus)
	dWM7	Left putamen (lP), anterior parahippocampal gyrus (APHCG), left caudate (lC), frontal sub gyral (FSG)
	dWM8	Right thalamus (rT), right putamen (rP), right temporal sub gyral (rTSG), right pallidum (rPa)
	dWM9	Middle cerebellar peduncle (MCP)
	dWM10	Left posterior corona radiata (lPCR), left superior longitudinal fasciculus (lSLF)
	dWM11	Right inferior longitudinal fasciculus (rILF)

#### 3.1.2 FNCs using static and dynamic features in WM

**Figure 6:**
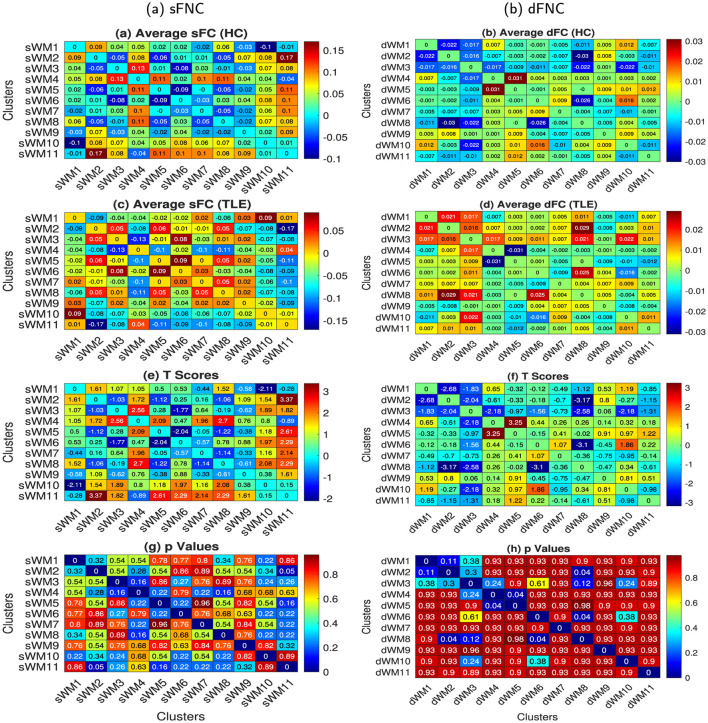
The FNC (static and dynamic) matrices were computed using the average time courses from the 11 WM networks obtained from the sFC and dFC clustering of ROIs. The 11 sFNs include the following networks: lPCR - lSLF - CC (splenium) (sWM1), FM - ACR - CC (genu) (sWM2), lHC - MB (sWM3), MCP - BS (sWM4), lT - lP - lC - lPa (sWM5), lTSG (sWM6), rT - rP - rC - rPa (sWM7), APHCG (sWM8), SCR - CC (body, left splenium) - rPCR (sWM9), rILF (sWM10), and lILF (sWM11). The 11 dFNs include the following networks: lILF (dWM1), lTSG - lT - lPa - lH (dWM2), CC (splenium) - BS (dWM3), CC (body) - SCR - rSLF (dWM4), FM - ACR - MFG (dWM5), cingulum (hippocampus) (dWM6), lP - APHCG - lC - FSG (dWM7), rT - rP - rTSG - rPa (dWM8), MCP (dWM9), lPCR - lSLF (dWM10), and rILF (dWM11) in WM. The left and right columns illustrate the sFNC and dFNC, respectively. From top to bottom, the figures illustrate the following: subjects' averaged sFNCs and dFNCs (after Fisher's Z-score transformation, and gender and age effects regressed out from FCs) of **(a, b)** HC and **(c, d)** TLE subject groups, respectively. **(e, f)** t-scores and **(g, h)** *p*-values of the two-sample t-tests between HC and TLE after FDR correction with the Benjamini and Hochberg method. We did not observe any significant differences between the lTLE and rTLE cohorts using both the sFC and dFC.

Using sFNs and static features from WM regions, we investigated network connections and identified static connections that significantly differ in TLE compared to HC, as discussed in Section 2.5.2. These connections are demonstrated in Figures 6a, c, e, g. The averaged network-based sFC for the HC and TLE groups is shown in Figures 6a, c, respectively. In Figures 6e, g, t-scores and their corresponding *p*-values of the two-sample *t*-tests are illustrated. It can be observed that the connectivities between FM - ACR - CC(genu) and lILF (sWM2 - sWM11) exhibit high t-scores with low p-values (*p* < 0.05), indicating significant alterations in TLE from HC (Figures 6e, g). The sFC between the networks decreased by 0.34 in TLE. No significant connections were observed between the HC and lTLE/rTLE, and lTLE and rTLE groups; the results have been demonstrated in Supplementary material. Diverse changes in connectivity between dFNs were investigated, as discussed in Section 2.6.2, and dynamic connections that were significantly altered in TLE from HC are demonstrated in Figures 6b, d, f, h. The averaged dFNC for the HC and TLE groups is shown in Figures 6b, d, respectively. Two-sample *t*-tests were conducted between groups, and t-scores and their corresponding *p*-values are illustrated in Figures 6f, h, respectively. It can be observed that the dFC between the network CC(body) - SCR - rSLF and FM - ACR - MFG (dWM4 - dWM5) decreased significantly (*p* < 0.05) by 0.09 in TLE from HC. The connectivity between the lTSG - lT - lPa - lH and rT - rP - rTSG - rPa networks (dWM2 - dWM8) exhibited low t-scores with low p-values (*p* < 0.05), indicating significant alterations in TLE from HC (Figures 6f, h). The dFC between the networks increased by 0.06 in TLE. The dynamic connectivity between the Cingulum (hippocampus) and rT - rP - rTSG - rPa networks (dWM6 - dWM8) increased significantly by 0.051 (*p* < 0.05). We could not find any significant dFNC changes between the HC and lTLE/rTLE, or between lTLE and rTLE groups; the results have been demonstrated in Supplementary material.

#### 3.1.3 Static and dynamic functional connectivity strengths of networks in WM

**Figure 7:**
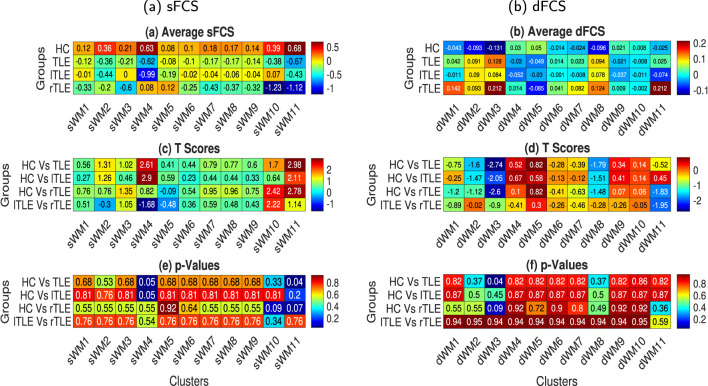
The average static and dynamic functional connectivity strength (sFCS and dFCS) for the 11 sFNs [lPCR - lSLF - CC(splenium) (sWM1), FM - ACR - CC(genu) (sWM2), lHC - MB (sWM3), MCP - BS (sWM4), lT - lP - lC - lPa (sWM5), lTSG (sWM6), rT - rP - rC - rPa (sWM7), APHCG (sWM8), SCR - CC (body, left splenium) - rPCR (sWM9), rILF (sWM10), and lILF (sWM11)) and 11 dFNs [lILF (dWM1), lTSG - lT - lPa - lH (dWM2), CC (splenium) - BS (dWM3), CC (body) - SCR - rSLF (dWM4), FM - ACR - MFG (dWM5), cingulum (hippocampus) (dWM6), lP - APHCG - lC - FSG (dWM7), rT - rP - rTSG - rPa (dWM8), MCP (dWM9), lPCR - lSLF (dWM10), and rILF (dWM11)] in WM. The left and right columns illustrate the sFCS and dFCS, respectively. **(a, b)** Subject-averaged sFCS and dFCS of the various networks (left to right) in different groups (top to bottom), respectively. To compute the FCS, all sFCs and dFCs were Fisher's Z-score transformed, and the effects of gender and age were regressed out. **(c, d)** t-scores and **(e, f)** corresponding *p*-values obtained from two-sample *t*-tests between different subject groups (top to bottom) and various sFNs and dFNs, respectively (left to right).

Static functional connectivity strengths were computed for every sFN as described in Section 2.5.3. Average sFCS for four cohorts (HC, TLE, rTLE, and lTLE) and 11 sFNs are illustrated in Figure 7a. Two-sample *t*-tests were conducted, and the resulting t-scores and corresponding *p*-values (rounded to two decimal places) are shown in Figures 7c, e, respectively. lILF (sWM11) exhibits a significant (*p* < 0.05) alteration in TLE from HC by decreasing its average sFCS by a significant extent of 1.35. Other sFNs couldn't find any significant dysfunction among the remaining group pairs. Total variation in FCs was also computed for every dFN as described in Section 2.6.3 using the dFCS measure. Average dFCS for four cohorts (HC, TLE, rTLE, and lTLE) and 11 dFNs are illustrated in Figure 7b. Two-sample *t*-tests were conducted, and the resulting t-scores and corresponding *p*-values are demonstrated in Figures 7d, f, respectively. Group pairs are demonstrated from top to bottom, and FNs are shown from left to right in Figures 7c–f. The CC(splenium)-BS (dWM3) network exhibited a significant (*p* < 0.05) alteration in TLE compared to HC with an increased dFCS of 0.26. We could not find any other dFNs significantly dysfunctioning among the remaining group pairs.

#### 3.1.4 Dependency between clinical features and static and dynamic FNCs

**Figure 8:**
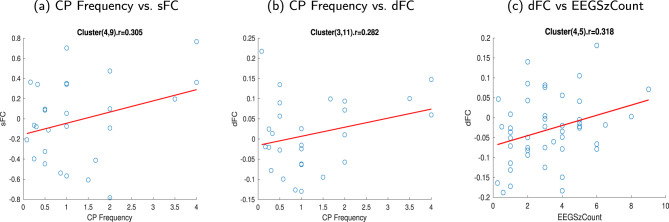
Scatter plot showing the relationship between FC and clinical features of TLE subjects. **(a)** Correlation (0.305) between the sFNC and the monthly frequency of complex partial seizures (CP Freq) for the sFN pair MCP - BS and SCR - CC (body, splenium) - SLF - rPCR (sWM4 - sWM9), **(b)** Correlation (0.282) between the dFC and the monthly frequency of complex partial seizures (CP Freq) for the dFN pair CC(splenium)-BS and rILF (dWM3 - dWM11), **(c)** Correlation (0.318) between the dFNC and the number of seizures captured (EEGSzCount) for the dFN pair CC(body) - SCR - rSLF and FM - ACR - MFG (dWM4 - dWM5). **(a)** CP Frequency vs. sFC. **(b)** CP Frequency vs. dFC. **(c)** dFC vs. EEGSzCount.

The monthly frequency of complex partial seizures (CP Freq) and the number of seizures captured (EEGSzCount) in subjects with TLE were correlated with the sFNCs and dFNCs, respectively. Comparatively, high correlations were observed between the sFNC and CP Freq for the sFN pair MCP - BS (sWM4) and SCR - CC (body, splenium) - SLF - rPCR (sWM9) (Figure 8a). The correlation value for this connection was 0.305 (p corrected = 0.20). For dFNC, a high correlation (0.282, p corrected = 0.15) was observed in CP Freq between the CC (splenium) - BS (dWM3) and rILF (dWM 11) networks (Figure 8b). dFNC and EEGSzCount showed a high correlation (0.318, p corrected = 0.032) between the dFN pair CC (body) - SCR - rSLF (dWM4) and FM - ACR - MFG (dWM5), as observed in Figure 8c. The dataset included information on the “number of current anticonvulsant medications,” “Age at the onset of the first seizure,” and “Age at the onset of recurring seizures” used by individuals with TLE. The current number of anticonvulsant medications was classified into 4 categories from 1 to 4. As the reviewer suggested, we performed a correlation analysis with this, with different resting state network connectivities (static and dynamic), and did not find any significant correlations. We also performed a correlation analysis between disorder duration from the onset of the first seizure and the onset of the recurring seizure with the FNC. The disorder durations were obtained by subtracting the current age from the “Age at the onset of the first seizure” and “Age at the onset of the recurring seizure,” respectively. We observed no significant relation of the FNC with the number of current anticonvulsant medications, but we observed a negative correlation of the FNC with the disorder durations (Supplementary Figures 19–27). All the FCs and clinical variables are shown after outlier elimination. Data values that were more than three scaled median absolute deviations were considered outliers.

## 4 Discussion

In this study, we obtained resting-state functional networks by clustering resting-state fMRI data based on static and dynamic functionality within WM and subcortical GM regions. Using the average time courses for the various WM parcelations, both static and dynamic FC were computed. Static FC assumes that the FCs are stationary and estimates the time-averaged FC over the entire scan. In contrast, the dFC assumes that these connections are non-stationary and captures temporal variations of FC at shorter time windows, providing more insights into neuronal activity patterns (Hutchison et al., 2013; Allen et al., 2012; Reinen et al., 2018; Choe et al., 2017). The window-based dFC has been demonstrated to facilitate the identification of distinct brain states and explain the dynamics of brain network properties (Allen et al., 2012; Choe et al., 2017; Damaraju et al., 2014). The dFC-based clustering offers FNs within which the voxel connectivities change in a similar pattern, and the dFNCs elucidate how similarly connectivity of two networks changes or behaves. Our study first identifies the altered sFNCs in subjects with TLE. It then explores the dFNCs that change diversely between WM dFNs in subjects with TLE.

Some of the FNs derived from clustering the dFC were similar to those obtained from clustering the sFC (Figures 3, 4). The left and right ILF (sWM11, sWM10 in sFNs and dWM1 and dWM10 in dFNs, respectively), FM - ACR - CC (genu) (sWM2) and FM - ACR - MFG (dWM5), lPCR - SLF - CC (left splenium) (sWM 1) and lPCR - lSLF (dWM10), as well as MCP - MS (sWM4) and MCP (dWM9), showed significant similarity in both clustering methods. This similarity suggested that they were synchronous and changed in the same manner. Although many similarities were observed in the estimated networks derived from the clustering of static and dynamic FC, we also noted differences between the FNs, such as the rT - rP - rC - rPa, representing sWM7 in sFNs. In contrast, the corresponding right temporal subgyral area (dWM8) differed in the dFNs. In sWM9 of the sFNs, most of the corpus callosum (except the genu) was present, whereas only the corpus callosum (body) appeared in dWM4 of dFNs, and the corpus callosum (splenium) was present with a significant portion of the brain stem in dWM3 of the dFNs, implying that not all parts of the corpus callosums connectivity change in the same way. The left TSG represents sWM6 of the sFNs, but this same network appears alongside the left thalamus, pallidum, and hippocampus and represents dWM2 of the dFNs, implying that their connectivity changes in the same way.

By evaluating the FDR between FNs and comparing the control and TLE groups (Figure 5), we observed the highest alterations in WM when considering the static interaction between the FM - ACR CC (genu) and lILF (sWM2 - sWM11), providing an FDR value of 0.22. However, the highest distinguishability between the lTLE and rTLE was observed while considering the static interaction between the MCP - BS (sWM4) and rT - rP - rC - rPa (sWM4 - sWM7), as well as the MCP - BS and SCR - CC (body, splenium) - SLF - rPCR (sWM4 - sWM9), raising the FDR value to 0.41 and 0.24, respectively. The dFNs have also exhibited alterations between HC and TLE subjects in the dynamic interaction between CC (body) - SCR - rSLF and FM - ACR - MFG (dWM4 - dWM5), resulting in an FDR of 0.205. The alteration in lTLE from rTLE was found in the dynamic connections between the CC (splenium) - BS and rILF (dWM3 - dWM11) networks, with an FDR of 0.215. This implies that dynamic connectivity was altered in subjects with TLE. The WM regions in the superior and anterior corona radiata, inferior and superior longitudinal fasciculus, temporal subgyral areas, forceps minor, and brainstem are primarily responsible for these changes.

The sFNC reveals a significant decrease in connectivity between the FM - ACR - CC (genu) and lILF (sWM2 - sWM11) in TLE (Figures 6a, c, e, g), but there was no significant connection between the lTLE and rTLE. The ILF has a long-range tract in the temporal lobe, strongly associated with the WM pathway, connecting the occipital and temporal areas of the brain to the anterior temporal regions. Previous studies have indicated that the ILF may be vulnerable to pathological changes in subjects with TLE (Kreilkamp et al., 2017). Dynamic connections between the CC(body) - SCR - rSLF and the FM - ACR - MFG (dWM4 - dWM5) decreased significantly in subjects with TLE (Figures 6b, d, f, h). This decrease implies that this connection behaves diversely with lower variability in TLE. Conversely, the dynamic connections between the lTSG - lT - lPa - lH and rT - rP - rTSG - rPa (dWM2 - dWM8), cingulum(hippocampus), and rThalamus-rP-rTSG (dWM6 - dWM8) increased significantly, suggesting these two connections changed with higher variability in TLE than in HC. A previous study showed that WM regions exhibited greater dynamics in FC (Wang et al., 2021), and we observed that these dynamics were more pronounced for alterations in TLE, particularly in the anterior corona radiata, CC(body), and temporal subgyral (contralateral) areas. The GM subcortical regions, including the hippocampus, thalamus (contralateral), right putamen, and right pallidum, exhibited alterations in dynamic connectivities. The sFC-based analysis showed one significant connection between HC and TLE, whereas the dFC-based analysis revealed three significant connections associated with five networks: CC(body) - SCR - rSLF, FM - ACR - MFG, lTSG - lT - lPa - lH, rT - rP - rTSG - Pa, and cingulum (hippocampus). In both analyses, the FM-ACR was common and exclusively a WM network. Besides this network, other significant networks in the dFC analysis were associated with subcortical GM regions, highlighting the crucial role of subcortical networks in TLE (Pulsipher et al., 2007; Dreifuss et al., 2001; Dabbs et al., 2012). In a previous study, TLE was also shown to be associated with increased hippocampal connectivity involving the limbic network, frontal lobes, angular gyrus, basal ganglia, thalamus, brainstem, and cerebellum, while exhibiting reduced connectivity involving visual, somatosensory, auditory, primary motor, and precuneus regions (Haneef et al., 2014). Additionally, the discrepancy in the number of significantly different networks between the sFC and dFC-based analyses highlights the ability of dFC-based analysis to quantify additional variations in functional networks, particularly regarding temporal changes, compared to static functional networks (Hutchison et al., 2013). Consequently, the dFC analysis may indicate which WM regions are more susceptible to transient and temporal fluctuations in neuronal activity due to TLE, in comparison to healthy controls. This localization can subsequently allow for a better understanding of the etiology of TLE as a whole, with further exploration of the causes behind such altered connectivity serving as a potential biomarker for TLE diagnosis. However, despite these network differences between TLE and HC, the sFNC and dFNC did not exhibit significant alterations between lTLE and rTLE. Future studies that account for more rTLE subjects may discover differences between these and lTLE.

The investigation of the sFCS across different networks revealed that the total static functional connectivity of the lILF with other networks was significantly altered in subjects with TLE, suggesting that the overall connectivity of lILF with other networks decreased in TLE (Figures 7a, c, e). Similarly, the investigation using dFC explored that the CC(splenium) - BS network was significantly altered in TLE, indicating that the total dynamic connections of this network in comparison to all other networks may vary notably (Figures 7b, d, f).

The two clinical variables (CP Freq and EEGSzCount) of the subjects with TLE showed higher correlations in the WM regions where high alterations were exhibited (Figure 8). CP Freq exhibited a higher correlation with the sFC obtained from the interaction between the MCP - BS and SCR - CC (body, splenium) - rSLF (sWM4 - sWM9). A higher correlation was also found between the dynamic connection of CC (splenium) - BS and rILF (dWM3 - dWM11). EEGSzCount exhibited a higher correlation with dFC obtained from the interaction between CC (splenium) - BS and FM - ACR - MFG (dWM3 - dWM5). These observations suggest that the WM regions: ILF, the superior and anterior corona radiata, and the corpus callosum show changes in connectivity related to the clinical variables.

TLE is classified into two forms: (1) Mesial temporal lobe epilepsy (mTLE), the most common subtype of TLE, accounts for temporal lobe seizures and involves the medial or internal structure of the temporal lobe. In mTLE, seizure activity often originates from a hippocampal or parahippocampal focus (Jackson et al., 2005). (2) Neocortical TLE (nTLE), where the epileptic focus is located in the lateral temporal lobe neocortex, the outer part of the temporal lobe (Kennedy and Schuele, 2012; Tatum, 2012), represents about 10% of TLEs (Téllez-Zenteno and Hernández-Ronquillo, 2012). The location of any temporal lobe ictal onset is mostly unknown for the subjects in the ECP dataset; therefore, we explicitly did not categorize the TLE subjects as mTLE.

The physiological origins of the WM-BOLD response are still not clearly known. Therefore, methodological aspects like the respiration signal and head motion artifacts merit further investigation (Jiang et al., 2019b). fMRI signals are believed to come from post-synaptic potentials (mainly in GM) rather than action potentials (Logothetis et al., 2001; Smith et al., 2002). However, studies present the probable sources of hemodynamic fluctuations in the WM, including vasculature and perfusion, astrocytes, and a higher glia-to-neuron ratio (Harris and Attwell, 2012; Engl and Attwell, 2015), nitric oxide-producing neuronal activity, and the metabolic demands of spiking activities (Gawryluk et al., 2014; Grajauskas et al., 2019). Other findings also support the hypothesis that fluctuations in WM-BOLD signals represent tract-specific responses to neuronal activity and hemodynamic responses to short stimuli (Gore et al., 2019). It has been demonstrated that the WM provides an intrinsic functional organization composed of interacting long-distance WM tracts (Peer et al., 2017; Concha et al., 2009). The studies also demonstrated that these networks were highly correlated to the resting state-gray matter (rs-GM) networks. Ding and colleagues have shown strong FC between WM tracts and cortical regions in the resting state, and the FC between specific regions was enhanced by specific functional loading (Ding et al., 2018). We found evidence in support of our hypothesis that the static and dynamic characteristics of brain activity are altered differently in deep temporal and extra-temporal regions.

The choice of window length for dFC computation is always a challenge. If the sliding window is too short, it may produce spurious values; however, if it is too long, it may overlook crucial dynamics. In this study, the window length used to demonstrate the dFC-based clusters and dFC-based FDR was 40 TR. This length was based on an earlier study (Laufs et al., 2014). Different window lengths can be used to analyze robustness.

We did not include any structural or behavioral information, such as IQ and handedness, both of which can influence alterations in functional connectivity due to their impact on the brain. While the diagnosis of epilepsy syndromes relies on multidisciplinary expertise and may slightly affect our findings, we believe that our primary results regarding group differences (HC vs. TLE) would remain unchanged. We have demonstrated the connectivity (both static and dynamic) between functional networks in WM; however, the interaction between GM and WM has not yet been characterized. In the future, we will explore this interaction.

This methodology could serve as a framework to identify patterns of altered connectivity for further studies on other neurological disorders, potentially expanding the scope of network analysis in clinical research. Our study presented the altered FC (both dynamic and static) in WM regions at the FN level. Our results indicate that the dynamic characteristics in WM matter may reveal alterations in cohorts with TLE.

## Data Availability

The original contributions presented in the study are included in the article/Supplementary material, further inquiries can be directed to the corresponding authors.

## Footnotes

^1^ https://osf.io/exbt4/

^2^ www.fil.ion.ucl.ac.uk/spm
