# Supplementary material for: Aberrant white matter and subcortical gray matter functional network connectivity associated with static and dynamic characteristics in subjects with temporal lobe epilepsy
Source: Front Neurosci. 2025 May 14;19:1571682. doi: 10.3389/fnins.2025.1571682 (PMC12118124; doi:10.3389/fnins.2025.1571682)
Supplement: Supplementary file 1 [file Data_Sheet_1.docx]

**Supplementary material for the manuscript ID 1571682:**

**Aberrant White Matter and Subcortical Gray Matter Functional Network Connectivity Associated with Static and Dynamic Characteristics in Subjects with Temporal Lobe Epilepsy**

Sukesh Kumar Das(skd54@njit.edu), George B Hanna (gbh6@njit.edu), Hai Sun (hs925@rwjms.rutgers.edu), Bharat B Biswal1([bharat.biswal@njit.edu](mailto:bharat.biswal@njit.edu))

1. **Demographic information of the Epilepsy connectome project (ECP) dataset:**

The number of healthy control (HC) males and females was 21 and 30, respectively. The number of individuals with temporal lobe epilepsy (TLE), male and female, was 26 and 26, respectively. Histograms of the ages of the HC and TLE groups have been demonstrated in Fig.1 and Fig.2, respectively. The mean and standard deviation of the ages of the HC and TLE groups are 34.41$\pm$11.10 and 37.28$\pm$12.16, respectively. In our experiments, the age and gender effects were regressed out from all functional connectivities (sFC/dFC) to derive the functional networks (clusters) and from the functional network connectivities (FNCs) (static or dynamic).

| 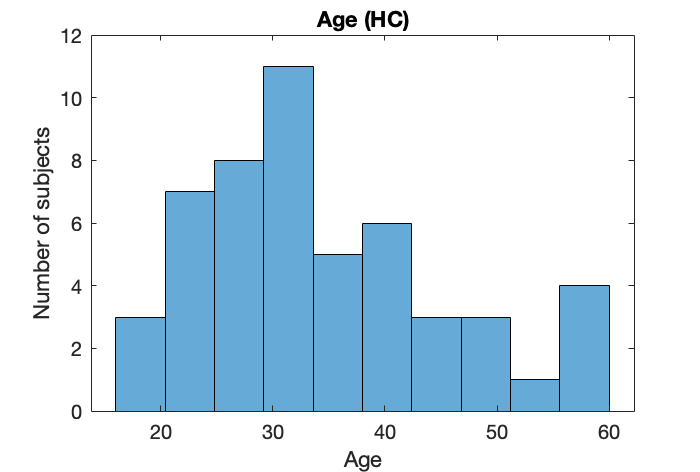 | 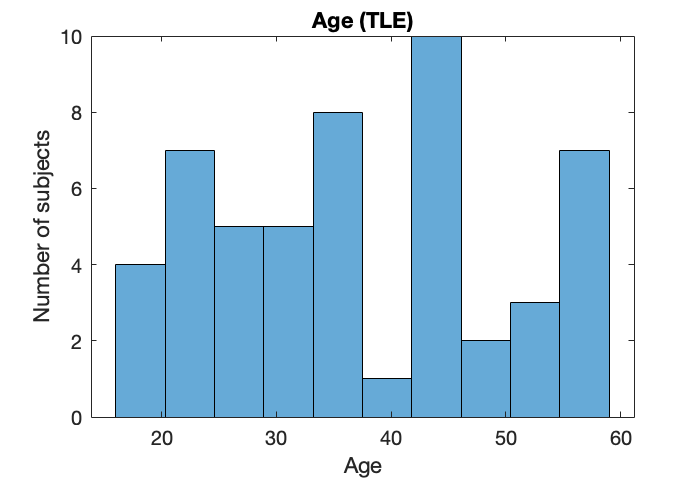 |
| --- | --- |
| Figure 1: Histogram of the ages of the healthy control | Figure 2: Histogram of the ages of the individuals with TLE |

The modified Edinburgh handedness quotient (MEHQ), ranging from -100 to 100, is demonstrated in the histograms of the HC and TLE groups in Fig.3 and Fig.4, respectively. If the value of the MEHQ is -100, then the subject is strongly left-handed, and if it is +100, then the subject is strongly right-handed. The mean and standard deviation of the MEHQ of the HC and TLE groups are 82.69$\pm$31.23 and 77.04$\pm$38.85, respectively.

| 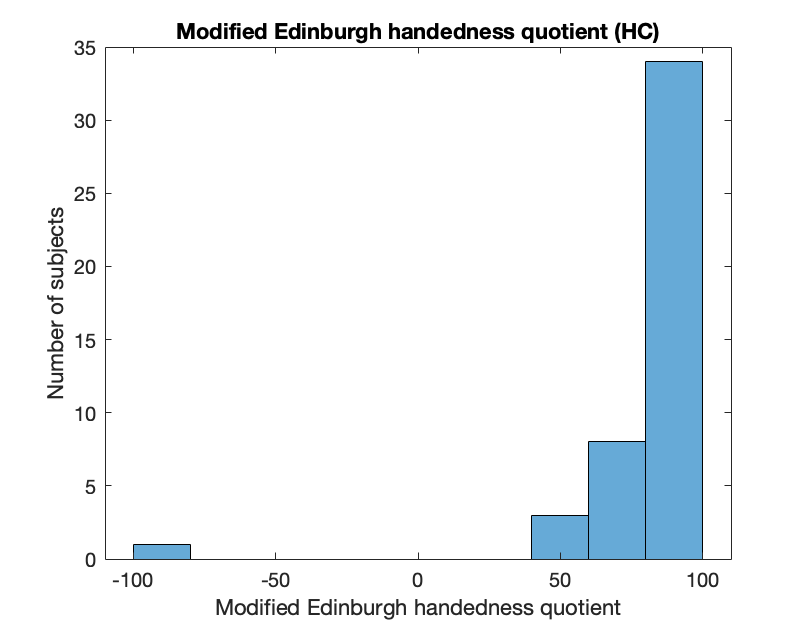 | 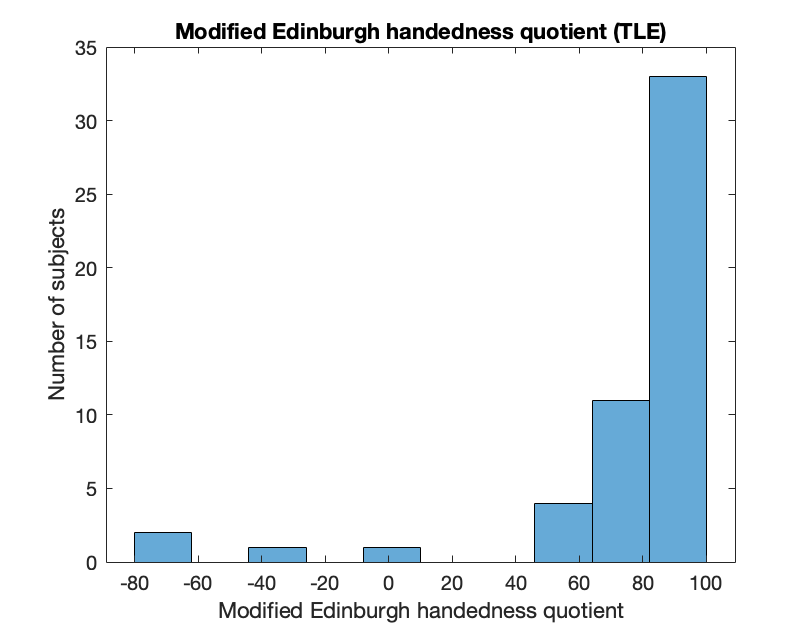 |
| --- | --- |
| Figure 3: Histogram of the MEHQ of the healthy control | Figure 4: Histogram of the MEHQ of the individuals with TLE |

1. **Fishers’ discriminant ratio (FDR) between HC and left/right TLE of WM network pairs:**

Using functional network connectivity (FNC), we also conducted FDR analysis to investigate the separability between HC and TLE, HC and left/right TLE and left TLE (lTLE) and right TLE (rTLE) and FDR matrices are demonstrated in Figs 5-12. We observed higher FDR values (0.4, Fig. 10) between lTLE and rTLE in the network pair sWM4 - sWM7 compared to the FDR values between HC and TLE, implying greater separability between lTLE and rTLE. In contrast, the dFNC-driven FDR exhibited higher values between HC and rTLE (0.43, Fig. 11), suggesting more dynamic changes between HC and rTLE for network pair dWM1 – dWM2. Interestingly, we also observed differences between HC and rTLE using dFC.

| 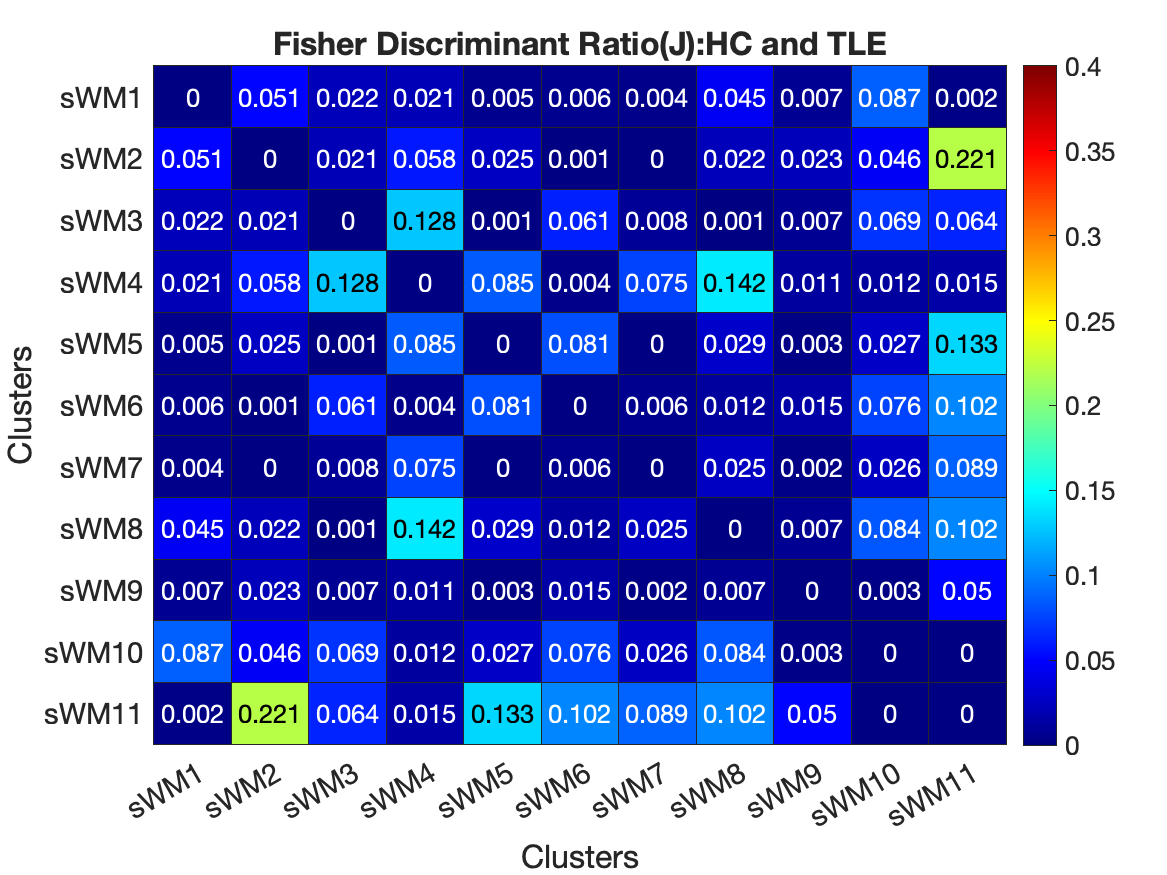 | 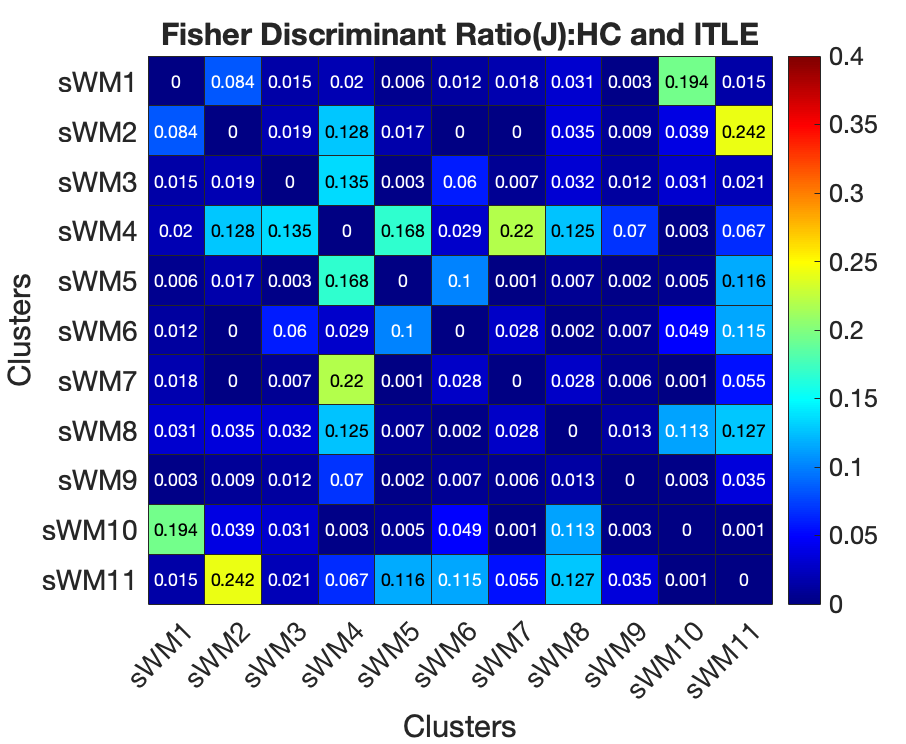 |
| --- | --- |
| Figure 5: FDR matrices between HC vs. TLE using the sFC for 11 FNs | Figure 6: FDR matrices between HC vs. lTLE using the sFC for 11 FNs |
| 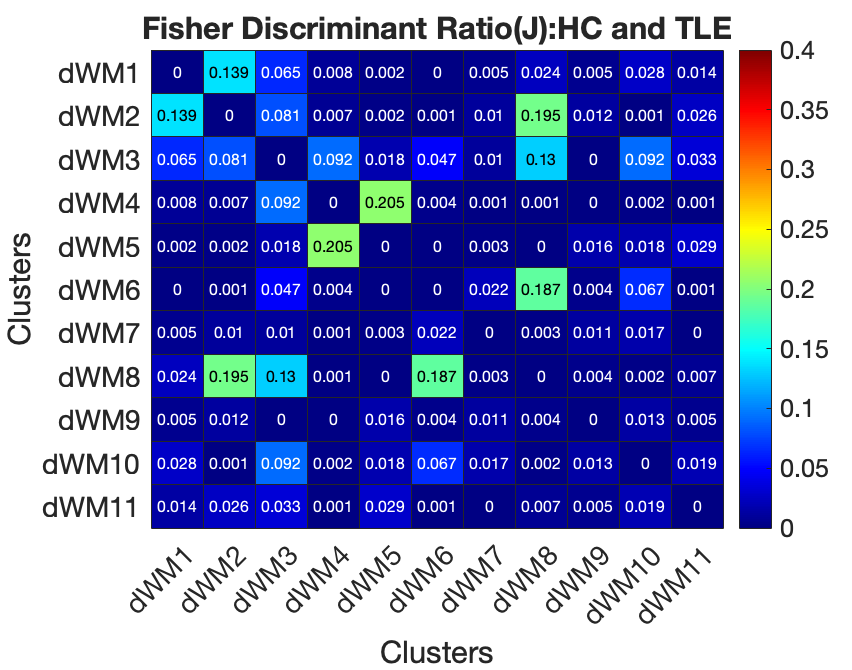 | 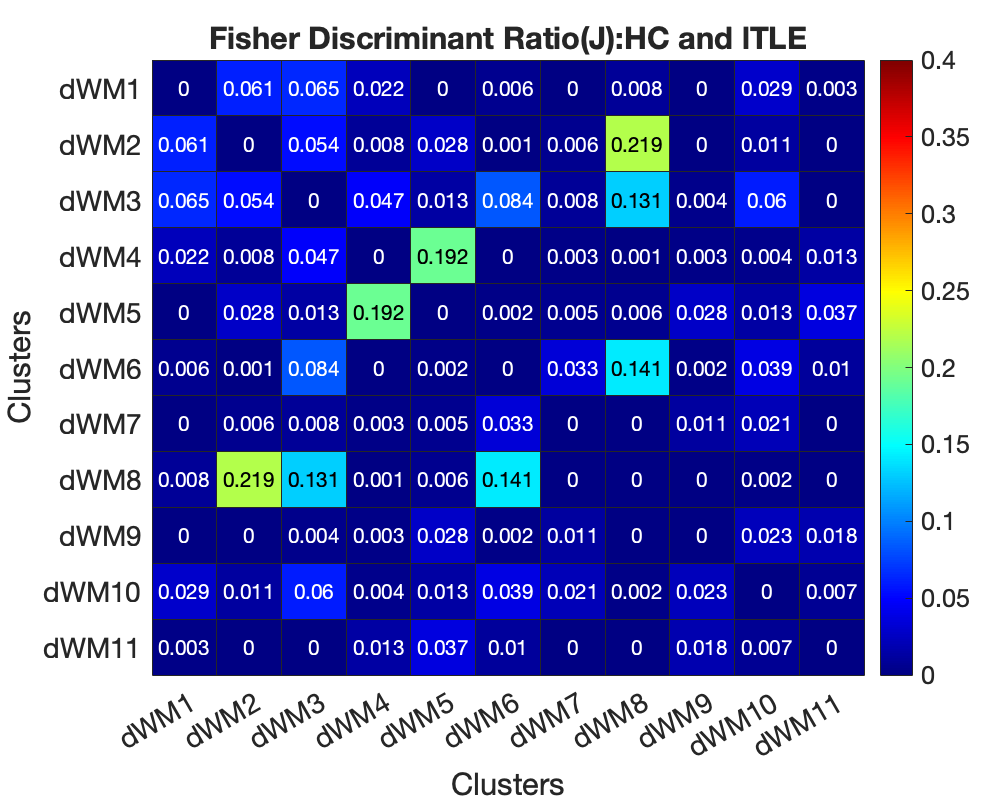 |
| Figure 7: FDR matrices between HC vs. TLE using the dFC for 11 FNs | Figure 8: FDR matrices between HC vs. lTLE using the dFC for 11 FNs |
| 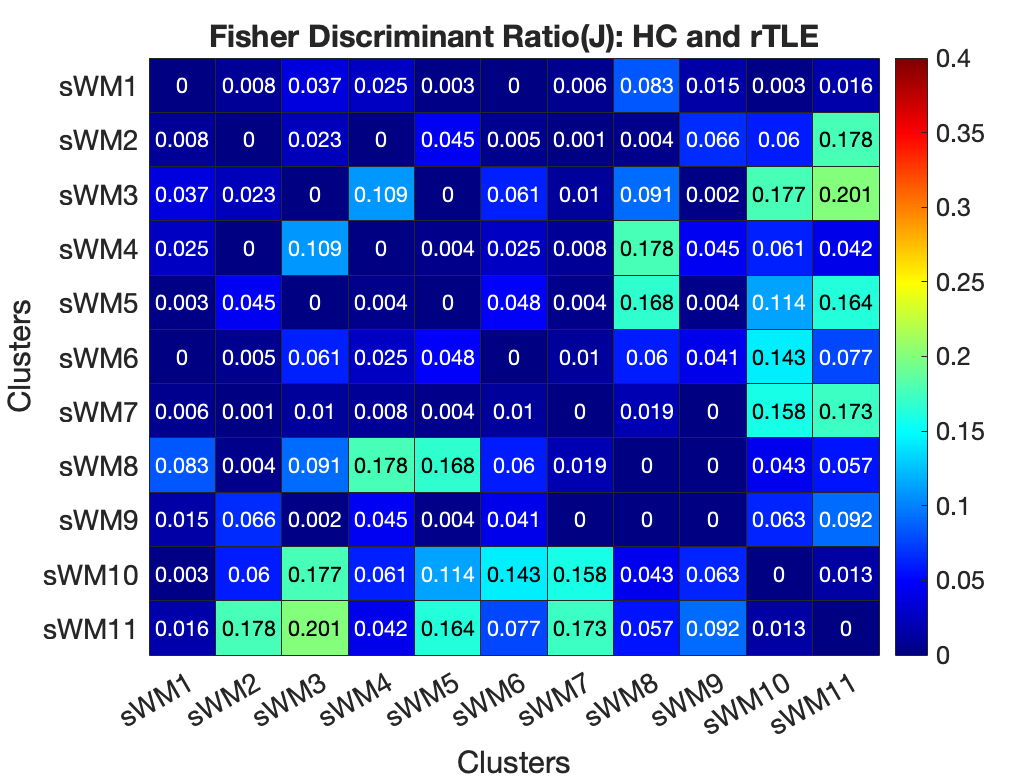 | 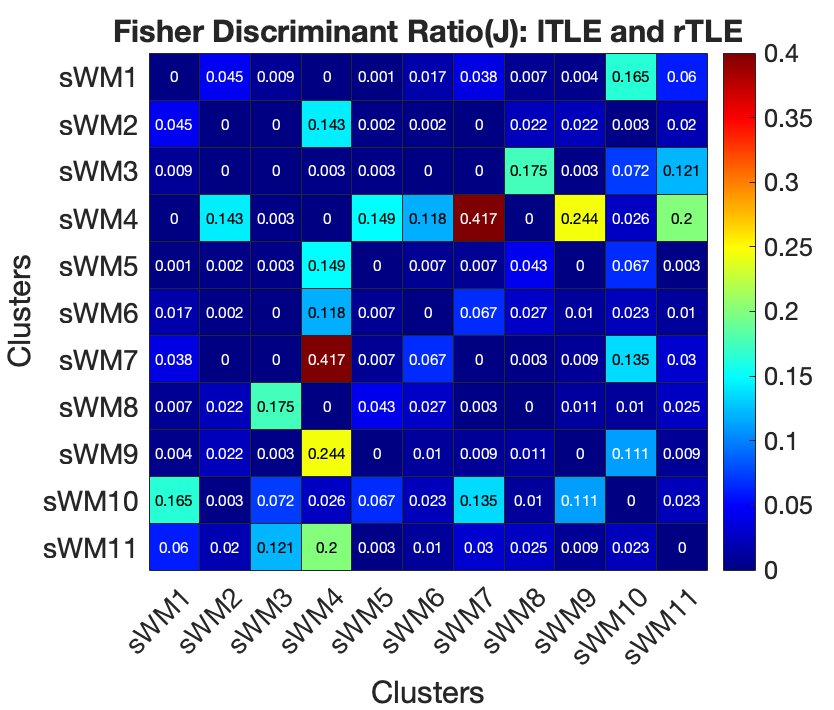 |
| Figure 9: FDR matrices between HC vs. rTLE using the sFC for 11 FNs | Figure 10: FDR matrices between lTLE vs. rTLE using the sFC for 11 FNs |
| 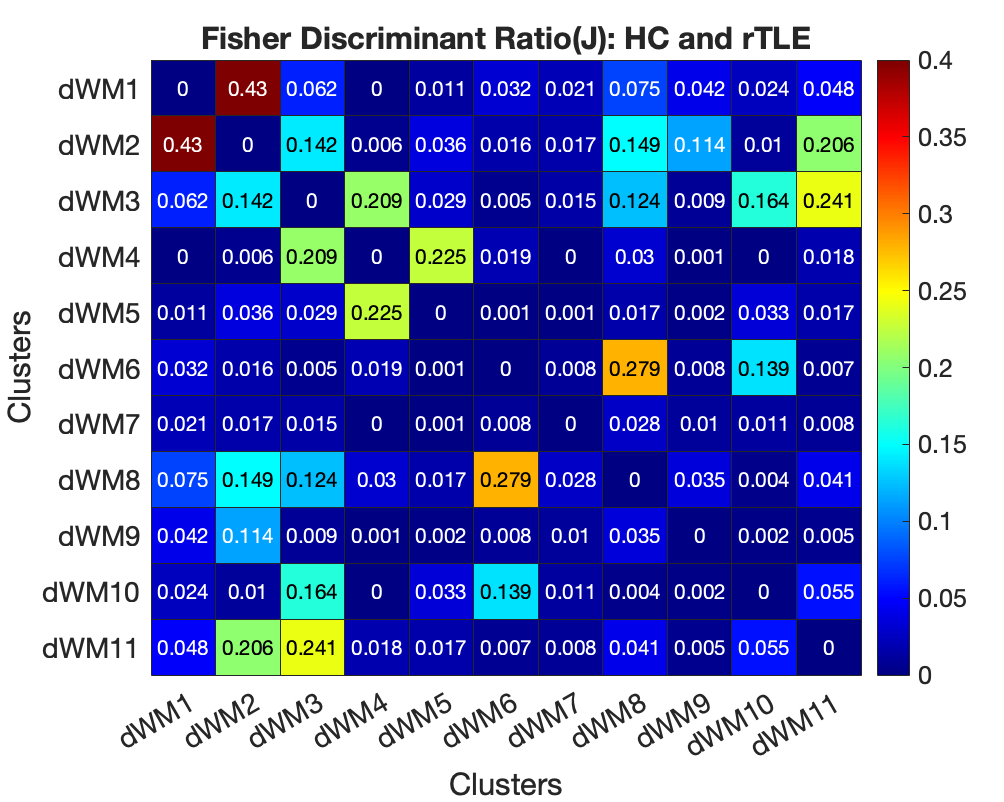 | 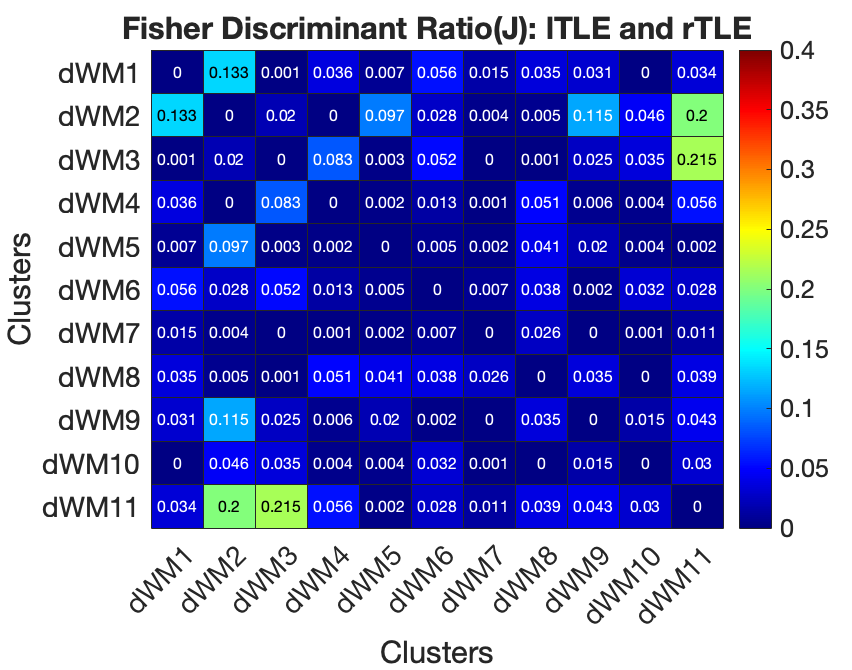 |
| Figure 11: FDR matrices between HC vs. rTLE using the dFC for 11 FNs | Figure 12: FDR matrices between lTLE vs. rTLE using the dFC for 11 FNs |

1. **White matter (WM) Functional network connectivity analysis using two sample t-test between healthy control (HC) and individuals with temporal lobe epilepsy (TLE):**

We conducted two sample t-tests on FNC (dynamic and static) between HC and lTLE/rTLE and between lTLE and rTLE as well and the average FNC, t-scores and corresponding corrected p-values are demonstrated in Figs. 13-18. However, after conducting the two-sample t-tests, we did not observe any significant differences between HC and lTLE/rTLE or between lTLE and rTLE in any of the cases (sFC or dFC-based).

| 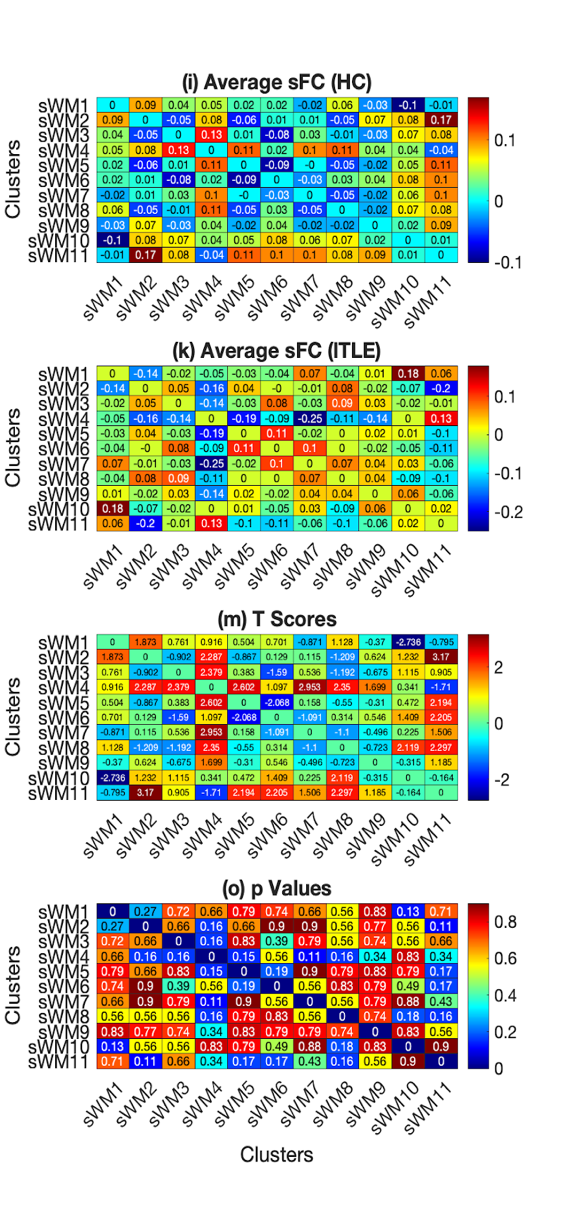 | 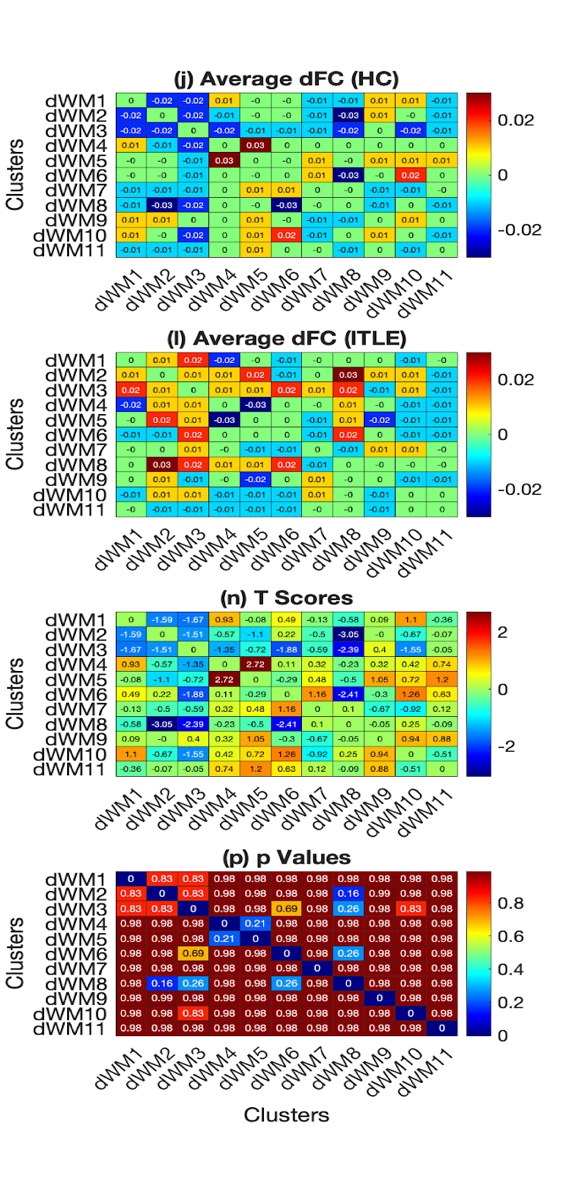 |
| --- | --- |
| Figure 13: (i) Average static FNC of HC, (k) average static FNC of lTLE, (m) t-scores after two-sample t-tests, and (o) p-values of the two-sample t-tests between HC and lTLE after *FDR* correction with the Benjamini and Hochberg method. | Figure 14: (j) Average dynamic FNC of HC, (l) average dynamic FNC of lTLE, (n) t-scores after two-sample t-tests, and (p) p-values of the two-sample t-tests between HC and lTLE after *FDR* correction with the Benjamini and Hochberg method. |
| 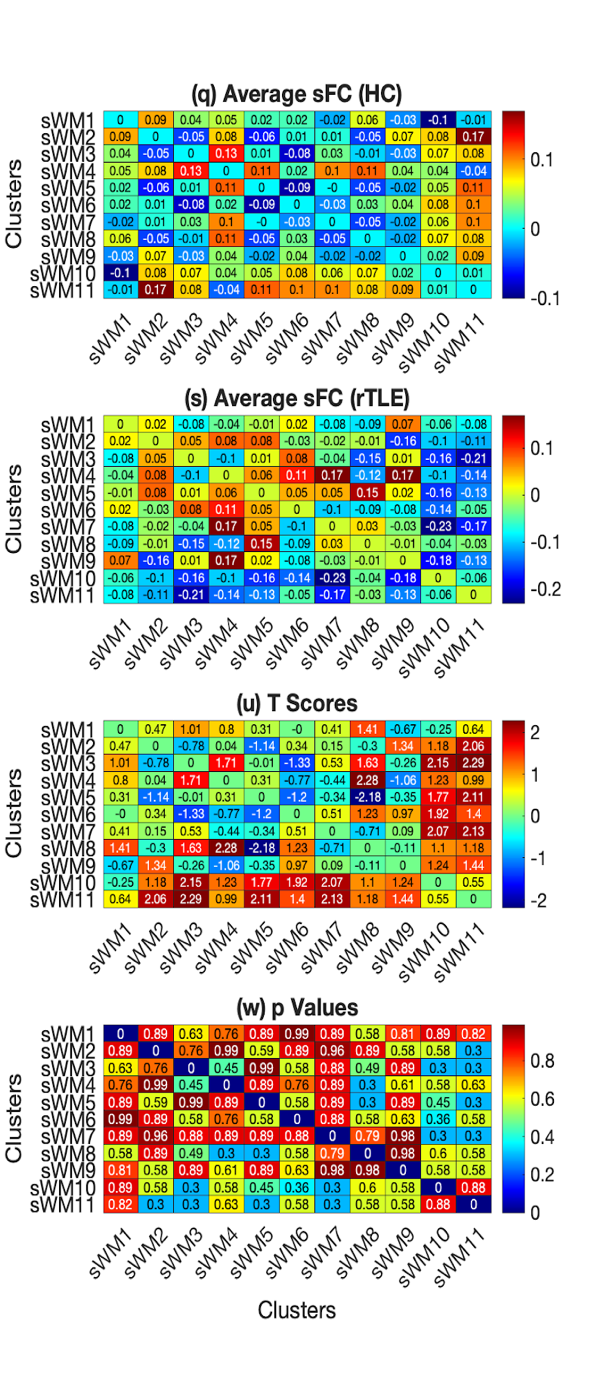 | 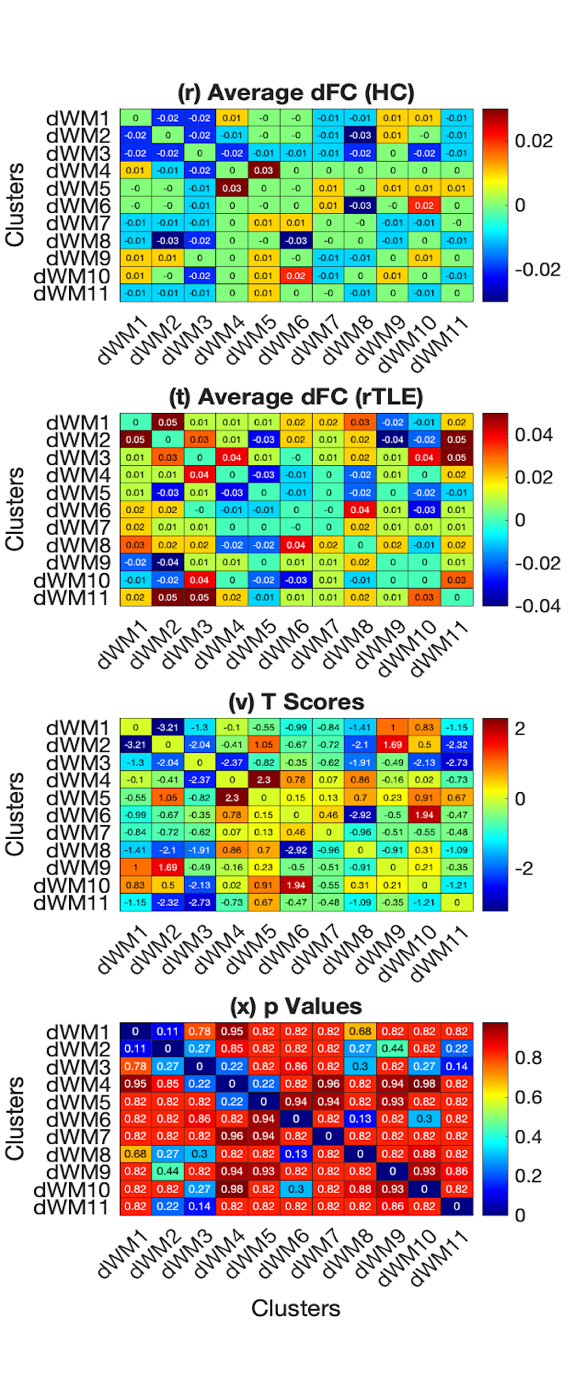 |
| Figure 15: (q) Average static FNC of HC, (s) average static FNC of rTLE, (u) t-scores after two-sample t-tests, and (w) p-values of the two-sample t-tests between HC and rTLE after *FDR* correction with the Benjamini and Hochberg method. | Figure 16: (r) Average dynamic FNC of HC, (t) average dynamic FNC of rTLE, (v) t-scores after two-sample t-tests, and (x) p-values of the two-sample t-tests between HC and rTLE after *FDR* correction with the Benjamini and Hochberg method. |
| 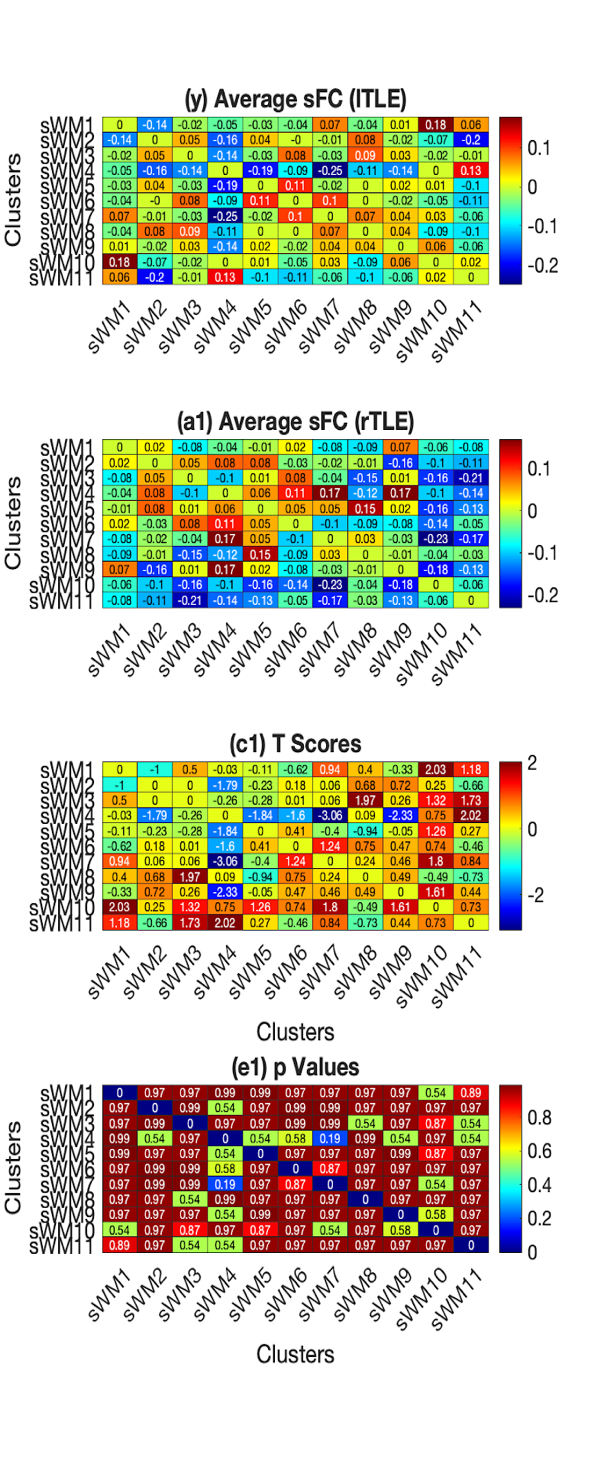 | 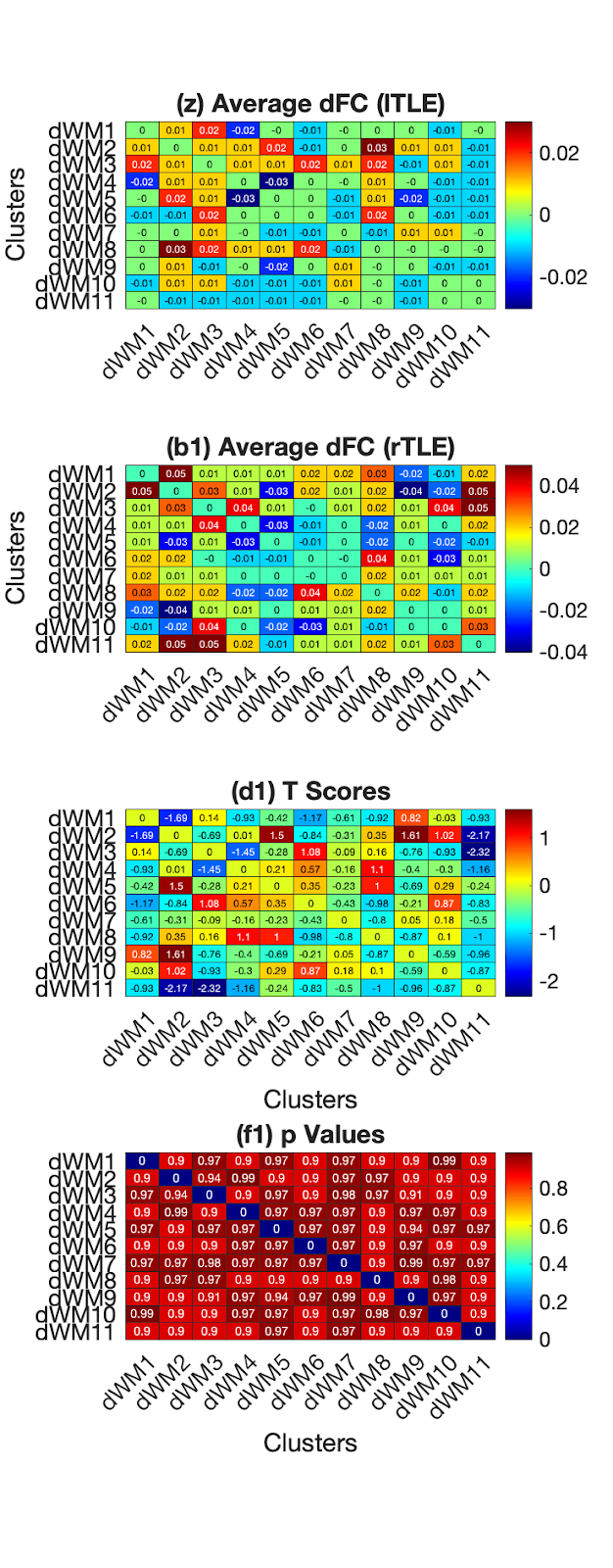 |
| Figure 17: (y) Average static FNC of lTLE, (a1) average static FNC of rTLE, (c1) t-scores after two-sample t-tests, and (e1) p-values of the two-sample t-tests between lTLE and rTLE after *FDR* correction with the Benjamini and Hochberg method. | Figure 18: (z) Average static FNC of lTLE, (b1) average static FNC of rTLE, (d1) t-scores after two sample t-tests, and (f1) p-values of the two-sample t-tests between lTLE and rTLE after *FDR* correction with the Benjamini and Hochberg method. |

1. **Relation between FNC and clinical features:**

The dataset included information on the ‘*number of current anticonvulsant medications’*, ‘*Age at onset of first seizure*’, ‘*Age at onset of recurring seizure*’ used by individuals with TLE. The current number of convulsant medication was classified into 4 categories from 1 to 4. As reviewer suggested, we performed a correlation analysis with this with different resting state network connectivities (static and dynamic) and did not find any significant correlations. We have also performed correlation analysis between disorder duration from the onset of the first seizure and the onset of the recurring seizure with the FNC. The disorder durations are obtained by subtracting the current age from the ‘Age at onset of first seizure’ and ‘Age at onset of recurring seizure’ respectively. We observed negative correlation of the FNC with the disorders durations (Figs.19-27).

| 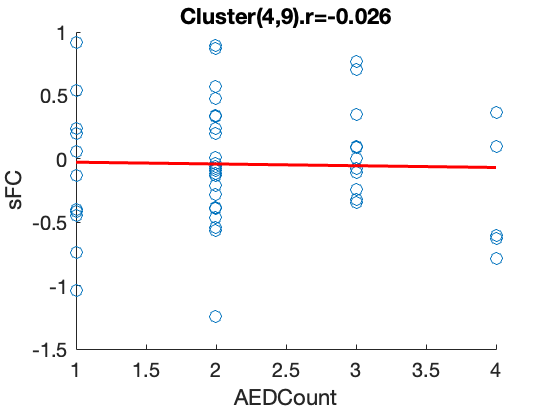 | 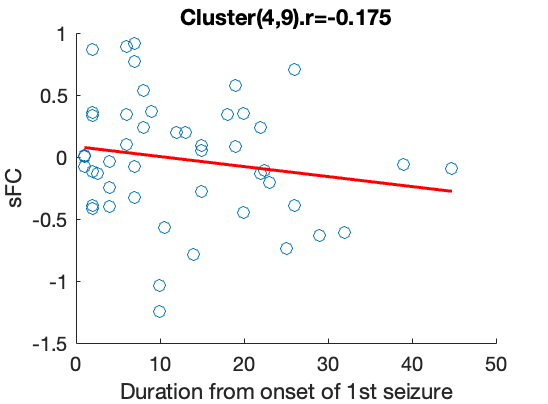 | 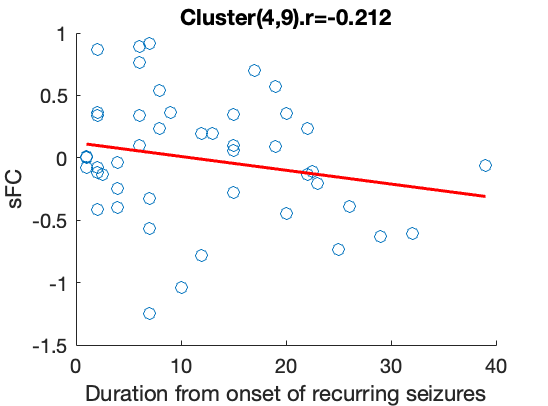 |
| --- | --- | --- |
| Figure 19: Correlation between static FNC (sWM4 - sWM9) and number of current anticonvulsant medications | Figure 20: Correlation between static FNC (sWM4 - sWM9) and disorder duration from the onset of the first seizure | Figure 21: Correlation between static FNC (sWM4 - sWM9) and disorder duration from the onset of the recurring seizure |
| 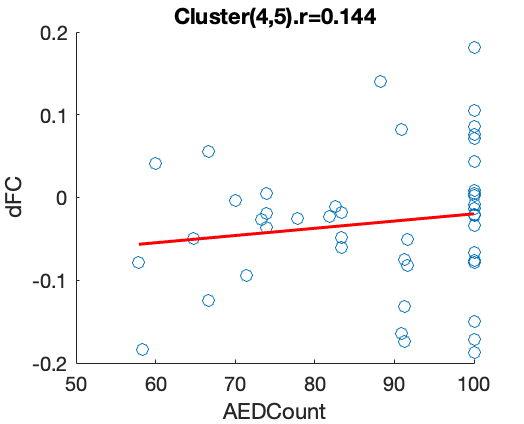 | 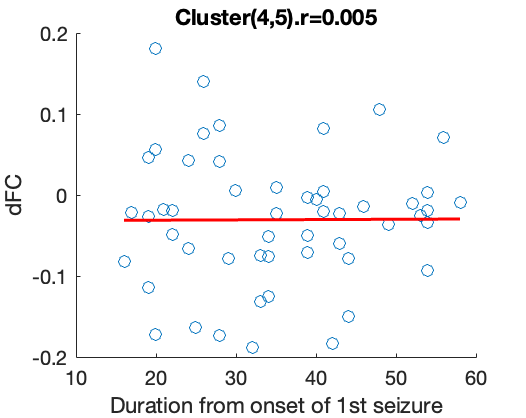 | 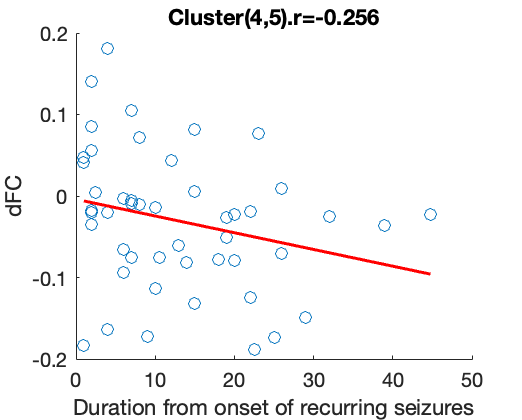 |
| Figure 22: Correlation between dynamic FNC (dWM4 - dWM5) and number of current anticonvulsant medications | Figure 23: Correlation between dynamic FNC (dWM4 - dWM5) and disorder duration from the onset of the first seizure | Figure 24: Correlation between dynamic FNC (dWM4 - dWM5) and disorder duration from the onset of the recurring seizure |
| 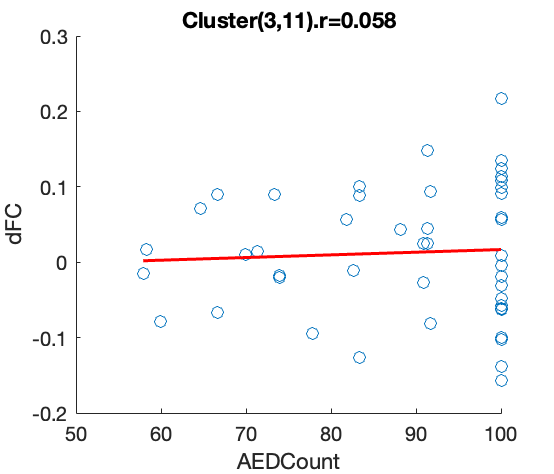 | 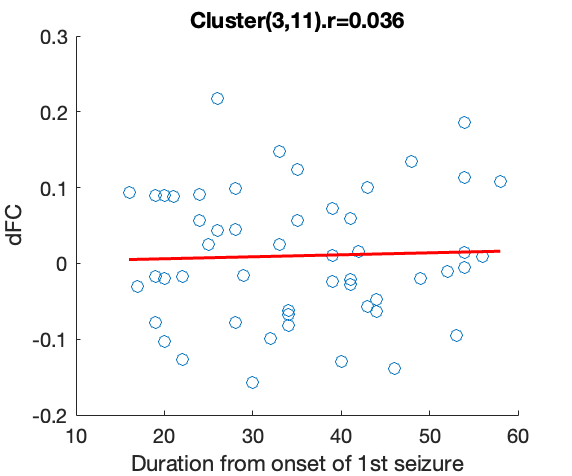 | 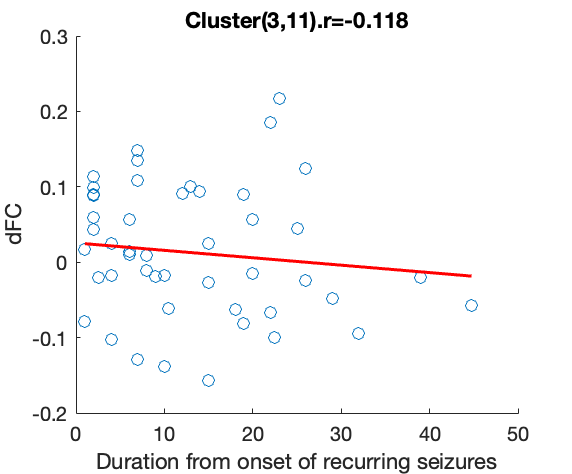 |
| Figure 25: Correlation between dynamic FNC (dWM3 - dWM11) and number of current anticonvulsant medications | Figure 26: Correlation between dynamic FNC (dWM3 - dWM11) and disorder duration from the onset of the first seizure | Figure 27: Correlation between dynamic FNC (dWM3 - dWM11) and disorder duration from the onset of the recurring seizure |
